# Motif models proposing independent and interdependent impacts of nucleotides are related to high and low affinity transcription factor binding sites in Arabidopsis

**DOI:** 10.3389/fpls.2022.938545

**Published:** 2022-07-28

**Authors:** Anton V. Tsukanov, Victoria V. Mironova, Victor G. Levitsky

**Affiliations:** ^1^Department of Systems Biology, Institute of Cytology and Genetics, Novosibirsk, Russia; ^2^Department of Plant Systems Physiology, Radboud Institute for Biological and Environmental Sciences (RIBES), Radboud University, Nijmegen, Netherlands; ^3^Department of Natural Science, Novosibirsk State University, Novosibirsk, Russia

**Keywords:** *de novo* motif search, heterogeneity of transcription factor binding sites, high and low affinity of transcription factor binding sites, standard and alternative motif recognition models, ChIP-seq data analysis

## Abstract

Position weight matrix (PWM) is the traditional motif model representing the transcription factor (TF) binding sites. It proposes that the positions contribute independently to TFs binding affinity, although this hypothesis does not fit the data perfectly. This explains why PWM hits are missing in a substantial fraction of ChIP-seq peaks. To study various modes of the direct binding of plant TFs, we compiled the benchmark collection of 111 ChIP-seq datasets for *Arabidopsis thaliana*, and applied the traditional PWM, and two alternative motif models BaMM and SiteGA, proposing the dependencies of the positions. The variation in the stringency of the recognition thresholds for the models proposed that the hits of PWM, BaMM, and SiteGA models are associated with the sites of high/medium, any, and low affinity, respectively. At the medium recognition threshold, about 60% of ChIP-seq peaks contain PWM hits consisting of conserved core consensuses, while BaMM and SiteGA provide hits for an additional 15% of peaks in which a weaker core consensus is compensated through intra-motif dependencies. The presence/absence of these dependencies in the motifs of alternative/traditional models was confirmed by the dependency logo DepLogo visualizing the position-wise partitioning of the alignments of predicted sites. We exemplify the detailed analysis of ChIP-seq profiles for plant TFs CCA1, MYC2, and SEP3. Gene ontology (GO) enrichment analysis revealed that among the three motif models, the SiteGA had the highest portions of genes with the significantly enriched GO terms among all predicted genes. We showed that both alternative motif models provide for traditional PWM greater extensions in predicted sites for TFs MYC2/SEP3 with condition/tissue specific functions, compared to those for TF CCA1 with housekeeping functions. Overall, the combined application of standard and alternative motif models is beneficial to detect various modes of the direct TF-DNA interactions in the maximal portion of ChIP-seq loci.

## 1. Introduction

Transcription factors (TFs) read out the gene regulatory nucleotide context (TF binding sites, TFBS) in a genome and subsequently initiate RNA synthesis (Lambert et al., 2018). Sequence motifs are short, recurring patterns in DNA that are presumed to have a biological function of sequence-specific binding sites (BSs) for certain TF (D'Haeseleer, 2006). TFs bind genomic DNA directly or indirectly, through cooperation with other TFs, or by modifying/remodeling chromatin proteins (Iwafuchi-Doi, 2019; Srivastava and Mahony, 2020). One of the most important tasks of molecular biology is to locate TFBSs genome-wide and thereby detect a TF's direct targets. Chromatin immunoprecipitation followed by massive sequencing (ChIP-seq) is a widely applied experimental technique to solve these problems (Johnson et al., 2007; Farnham, 2009; Park, 2009). Primary ChIP-seq data processing identifies genome loci, or peaks, in which a target TF binds DNA directly or indirectly through co-binding intermediary factors (Furey, 2012; Yu et al., 2021). The lengths of peaks comprise hundreds of base pairs, however, a TFBS usually does not exceed 20–25 base pairs in length (O'Malley et al., 2016; Kulakovskiy et al., 2018). Thus, at the second step in the ChIP-seq data processing, one searches for exact positions of sites in peaks. To date, many tools have been developed to solve this issue, the overwhelming majority of them are based on the motif model of position weight matrix (PWM; Stormo, 2000). The popular examples are STREME (Bailey, 2021) and HOMER (Heinz et al., 2010). Different implementations of the PWM model have been included in almost every pipeline of ChIP-seq data processing (Lloyd and Bao, 2019), despite that 20 years ago it was proved that this model was not quite correct (Benos, 2002; Bulyk et al., 2002).

On average, the PWM model detects reliable hits for about 60% of ChIP-seq peaks; this low estimate may be a consequence of (a) an indirect TF binding or (b) the disadvantage of PWM models in ignoring the dependencies of nucleotides occurrences in different site positions (Hunt et al., 2014; Gheorghe et al., 2019). The latter may negatively affect the recognition accuracy (Benos, 2002; Keilwagen and Grau, 2015). Therefore, alternative motif models took into account the dependencies between the nucleotides occurrences in distinct positions of a site (Mathelier and Wasserman, 2013; Gheorghe et al., 2019). The simplest alternative, the dinucleotide position weight matrix (diPWM), took into account the dependencies of adjacent positions (Zhang and Marr, 1993; Kulakovskiy et al., 2013). Dependencies of several close positions (“short-range”) were considered in more complicated “DNA shape” models (Zhou et al., 2013; Yang et al., 2014; Samee et al., 2019) and Markov chain models, e.g., BaMM (Siebert and Söding, 2016) and InMoDe (Eggeling et al., 2017). Application of these models revealed that BaMM might outperform PWMs (Siebert and Söding, 2016; Ge et al., 2021; Tsukanov et al., 2021), and InMoDe might predict TFBS of diverse structure (Eggeling, 2018). There were more general approaches to deduce both short- and long-range (arbitrary) dependencies within the motifs (Levitsky et al., 2007; Keilwagen and Grau, 2015). Earlier, we proposed the model SiteGA (Levitsky et al., 2007), which applied a genetic algorithm and deduced the discriminant function of frequencies of locally positioned dinucleotides within motifs. We experimentally proved the correctness of the SiteGA predictions for mammalian TFs FOXA2 and SF-1 and concluded that it might successfully complement traditional PWM models in ChIP-seq data analysis (Levitsky et al., 2014, 2016). Recently, we proposed the approach MultiDeNA that combined methodologically different *de novo* motif models for ChIP-seq data analysis (Tsukanov et al., 2021). Thus, we showed that alternative motif models might successively complement predictions of the standard PWM model. Obviously, the alternative models might find certain specific nucleotide contexts besides well-known PWM consensuses in ChIP-seq data.

Unfortunately, the standard PWM and its alternative models were not applied systematically to solve the problem of incomplete recognition of TFBS in ChIP-seq data. Hence, here we apply MultiDeNA (Tsukanov et al., 2021) both to deal with the structural heterogeneity of TFBSs and verify a larger portion of ChIP-seq loci using different motif models. To test the performance of this approach for plant TFs, we analyze over a hundred of available ChIP-seq datasets from Gene Transcription Regulation Database (GTRD) for *Arabidopsis thaliana* (Kolmykov et al., 2021). We performed a more detailed analysis of ChIP-seq datasets for CCA1, MYC2, and SEP3 TFs.

The first TF CCA1 (CIRCADIAN CLOCK ASSOCIATED1) from the Myb-like family is a key component of the circadian clock regulation. CCA1 is able to initiate and set the phase of clock-controlled rhythms (Nagel et al., 2015). Almost 90% of Arabidopsis transcripts cycle in at least one condition and most genes have peak expression at a particular time of day (Michael et al., 2008). The second TF MYC2 from the basic helix-loop-helix (bHLH) family is the master regulator of jasmonates-mediated responses, it activates the expression of the genes mediating plant defense against abiotic and biotic stress, including the synthesis of glucosinolates, terpenoids, and other specialized metabolites; MYC2 also plays a role in multiple developmental processes (Kazan and Manners, 2013; Schweizer et al., 2013). Jasmonates are plant hormones that regulate plant growth and development (Howe et al., 2018). The third TF SEP3 (SEPALLATA3) from the MIKC_MADS family is a key regulator of flower development (Smaczniak et al., 2012). In Arabidopsis, SEP3 drives the formation of distinct multimeric complexes important for floral organ identity (Immink et al., 2009).

We have confirmed that nucleotide context patterns respecting the motif models PWM, BaMM, and SiteGA possess only the moderate similarity, and various models define distinct weakly conserved sequences outside of the more conserved motif cores. Our analysis proposes that while the standard PWMs describe the position-specific conserved nucleotide context that is associated with sites of relatively higher affinity, the alternative models BaMM/SiteGA incorporate many dependencies of the various positions, which allows these models to achieve a more accurate representation of low affinity sites. A combination of methodologically various models takes into account substantially greater numbers of options for *in vivo* TF-DNA interactions; hence, the results of our analysis supported a notably larger portion of ChIP-seq loci with context-specific binding events.

## 2. Materials and methods

### 2.1. ChIP-seq data and motif models

For the analysis, we used ChIP-seq peak datasets for *A. thaliana* from GTRD (https://gtrd.biouml.org; Kolmykov et al., 2021). We included in the analysis the benchmark collection of 111 ChIP-seq datasets supported by additional input control experiments (Supplementary Table 1). For each dataset, we used in analysis 1,000 top-scoring peaks according to GTRD annotations of the MACS2 peak caller (Zhang et al., 2008). For *de novo* motif search we used the standard motif model PWM (STREME tool from the MEME suite version 5.4.1, https://meme-suite.org/meme/doc/download.html; Bailey, 2021), and two alternative models BaMM (https://github.com/soedinglab/BaMMmotif2, version 2; Siebert and Söding, 2016) and SiteGA (SiteGA package https://github.com/parthian-sterlet/sitega and Supplementary methods; Levitsky et al., 2007).

In *de novo* motif search, we took ChIP-seq datasets as the foreground datasets, and we compiled the background datasets from the randomly chosen sequences from the whole genome. The fractions of (A+T) nucleotides in the background sequences respect those in peaks with 1% precision; the foreground and background sequences possess the same distribution of lengths. We ensured that any background sequence did not possess even a partial perfect overlap with any sequence from the foreground dataset. We included the background generation program in the SiteGA package (see above). We used the following values of parameters in this program: (a) the maximal number of background sequences per one peak, the values 5 (PWM/BaMM) and 10 (SiteGA); in separate tests for three motif models, we changed this parameter from 1 to 10 and we confirmed that the performance estimates have remained almost the same; (b) the total number of attempts to get the background sequences from the genome per one foreground sequence, the value 500 (this restriction was required to fasten calculations for relatively rare peaks with extremely abnormal nucleotide content). Hence, the exact size of the background dataset several times exceeded the foreground dataset.

We used the cross-validation procedure to select parameters of models and to estimate the recognition performance of models. We selected model parameters within the following ranges: the motif lengths 8, 12, 16, and 20 bp for all models; the orders of the Markov model 1, 2, and 3 for BaMM; and the numbers of locally positioned dinucleotides (LPDs) 40, 60, 80, and 100 for SiteGA. For all other parameters of PWM STREME, BaMM, and SiteGA models, we applied their default recommended by developers values. We applied the 2-fold bootstrap cross-validation procedure, alternately using odd/even ranked peaks for the model training or estimating its accuracy. For each true positive rate (TPR, a fraction of the peaks containing at least one predicted motif in the foreground dataset), we calculated a false positive rate (FPR, a frequency of the predicted motifs in the background dataset) and drew the receiver operating characteristic (ROC) curve. To estimate the recognition performance of a model, we applied the measure partial area under ROC curve (pAUC). pAUC is defined as the sum of FPRs values below a certain threshold (McClish, 1989; Siebert and Söding, 2016). We employed the FPR threshold 1E-3 since the higher FPRs respected the recognition thresholds that commonly were not applicable in wide-genome analysis. We used the criterion of pAUC maximum for parameter setting for the models. We selected the thresholds for all three models as described earlier (Levitsky et al., 2019). Briefly, we defined an exhaustive list of the recognition scores through the computation of the expected recognition rates (ERRs) as the probabilities of the site prediction for the whole genome dataset of 1.5 kb long aligned upstream regions of *A. thaliana* protein coding genes. The recognition scores for a model were transformed to the uniform scale of ERRs, i.e., we compared the recognition scores of two distinct models that respected the same range of ERRs. We estimated each recognition threshold by a respective value of ERR and applied in analysis the stringent, medium, and mild thresholds corresponding to ERRs 1E-4, 2.5E-4, and 5E-4. Supplementary Figure 1 illustrates the parameters setting (the motif length, the number of LPDs) for the SiteGA model of the ChIP-seq dataset for MYC2 TF.

### 2.2. Combination of models by MultiDeNA pipeline

We used the Multiple *de novo* Analysis (MultiDeNA) pipeline (https://github.com/ubercomrade/MultiDeNA; Tsukanov et al., 2021), the software package for integrated application of distinct *de novo* motif models for ChIP-seq data analysis (Supplementary Figure 2). The pipeline takes several motif models to represent heterogeneous direct binding modes of a target TF and to achieve peaks classification according to presence/positioning of structurally distinct TFBS types. The pipeline includes the steps of accuracy assessment of models, their parameters estimation, models training, selection of applied recognition thresholds, scanning of peaks and their classification according to presence/co-localization of sites of separate models.

### 2.3. Statistical analysis

Data analysis and visualization were performed in the Python 3.8 language package numpy (Harris et al., 2020), pandas (McKinney, 2010), and matplotlib (Hunter, 2007). We used the Tomtom motif comparison tool to estimate the significance of motifs similarity (*p*-value < 0.05; Gupta et al., 2007). To apply this tool for a non-PWM motif model, we aligned its predicted sites and deduced a PWM motif, i.e., a position frequency matrix. We used the DepLogo R package (Keilwagen and Grau, 2015; Grau et al., 2019) to compare the traditional and alternative sequence logos. The alternative sequence logo visualizes the motif structure with respect to the dependencies between the sequence positions in predicted BSs. We applied the threshold of 0.05 for the DepLogo parameter of the mutual information value. To perform gene ontology (GO) enrichment analysis, we predicted the sites in the peaks with the medium thresholds (recognition scores respecting ERR ≤ 2.5E-4). Next, we mapped the peaks or their predicted sites within the entire genes and their upstream and downstream regions of 2,500 bp length. We considered in the analysis 27,206 protein-coding genes from the TAIR10 release of the *A. thaliana* genome. GO enrichment analysis was performed with the R package clusterProfiler (Yu et al., 2012). We used “biological processes” GO vocabulary only. We retained only GO terms with significant enrichment, the adjusted *p*-value of <0.05 (the significance of enrichment corrected for multiple testing). For each GO term, we computed the fold enrichment (see Table 1; Yu et al., 2012; Sherman et al., 2022) as the ratio between the frequency of predicted genes annotated with a GO term (Count_Obs+/Total_Obs) to the frequency of all genes annotated with this GO term (Count_Exp+/Total_Exp). We used the fold enrichment values to estimate the efficiency of the motif models in recognition of the genes specific for significantly enriched GO terms.

**Table 1:** 2 × 2 contingency table explaining GO enrichment analysis.

	**GO term present**	**GO term absent**	**Total**
Foreground (genes containing predicted sites of a model)	Count_Obs+	Count_Obs	Total_Obs
Background (all genes)	Count_Exp+	Count_Exp+	Total_Exp

## 3. Results

### 3.1. Analysis of the benchmark collection of *A. thaliana* ChIP-seq datasets

We compiled the benchmark collection of 111 *A. thaliana* ChIP-seq datasets for 52 target plant TFs from the GTRD (Kolmykov et al., 2021; see Supplementary Table 1 and Section 2). To ensure the reliability of this collection, we extracted known motifs for all target TFs from the CIS-BP database (Weirauch et al., 2014) and confirmed that for almost all datasets the known motifs of target TFs were notably enriched in respective ChIP-seq datasets (see Supplementary Table 1, we detected the fold enrichment above the thresholds 1 and 1.5 for 109 and 89 ChIP-seq datasets). We performed *de novo* motif search with three motif models: PWM (STREME; Bailey, 2021), BaMM (Siebert and Söding, 2016), and SiteGA (see Supplementary methods; Levitsky et al., 2007).

We computed the distributions of the frequency of predicted BSs according to the peak quality for PWM, BaMM, and SiteGA motif models for the benchmark collection of 111 *A. thaliana* ChIP-seq datasets, refer to Supplementary Figure 3. This analysis suggested that (a) the peaks with relatively high and low quality contained the proportional fractions of predicted BSs of individual models; (b) the total 1,000 top-scoring peaks, in general, were quite enough to detect the major structural types of motifs respecting three models.

**Figure 1:**
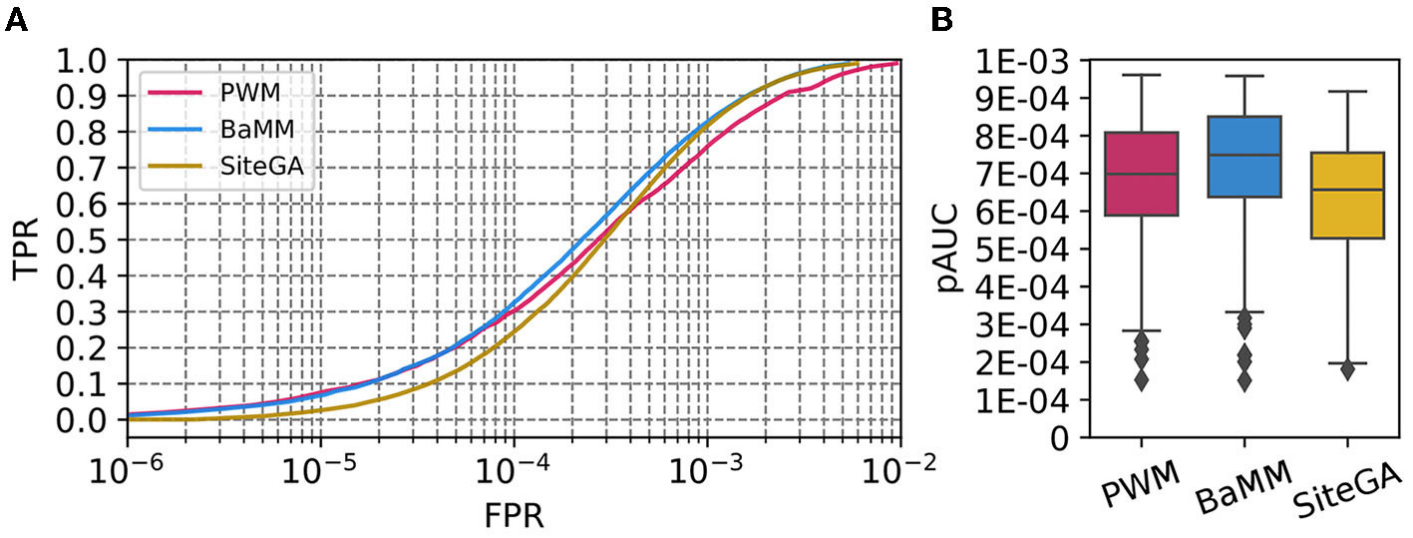
Performances of PWM, BaMM, and SiteGA motif recognition models for the benchmark collection of 111 *A. thaliana* ChIP-seq datasets. **(A)** ROC curves for the model's performance. We applied the bootstrap cross-validation procedure (see Section 2) and computed for each TPR (axis Y), the average FPRs (axis X) for the benchmark collection. **(B)** Boxplot distribution of the performance measure pAUC for three models. The boxplot presents the distributions of the *Q*_1_, *Q*_2_, and *Q*_3_ quartiles of the fractions of the peaks with recognized TFBS. Whiskers below/above the *Q*_1_/*Q*_3_ respect the minimum/maximum values if they were located within 1.5 interquartile ranges (*IQR* = *Q*_3_−*Q*_1_) from *Q*_1_/*Q*_3_, otherwise they are equal to {*Q*_1_−1.5*IQR}/{*Q*_3_+1.5*IQR}, respectively. In the latter case, we marked all other points as outliers.

For each dataset, we estimated the recognition performance of the models with the 2-fold cross-validation procedure and selected the parameters of the models (see Section 2, Supplementary Table 2). We constructed the ROC curves and evaluated the performance of the models with the measure pAUC (see Section 2). Figure 1A compares the distribution of ROC curves for the benchmark collection for three motif models. For each model, each value of axis Y for a certain TPR threshold (axis X) shows the average FPR of all datasets from the benchmark collection. We suggest that the scale of ERR values that defines the stringent, medium, and mild recognition thresholds (see Section 2), almost perfectly respects the scale of FPRs, that defines the X axis in a ROC curve. We can conclude that (a) for the stringent thresholds (FPR ≤ 1E-4), the SiteGA model yields a performance worse than those of PWM/BaMM; (b) for the medium thresholds (1E-4 < FPR ≤ 2.5E-4), an advantage of these two models is smaller, but BaMM is slightly better than PWM; (c) for the mild thresholds (2.5E-4 < FPR ≤ 5E-4), three models show similar performance, but BaMM is only slightly better than SiteGA, and SiteGA is better than PWM; (d) for extra mild thresholds (5E-4 < FPR ≤ 1E-3), BaMM and SiteGA are better than PWM. Figure 1B shows the boxplot distribution of pAUC values for the benchmark collection. The BaMM reveals the best pAUC (the median value 7.49E-4), PWM and SiteGA show the medium and worst thresholds (6.98E-4 and 6.57E-4, respectively). Overall, the performance of the models depends on the threshold selection: (a) the stringent or medium thresholds support the performance of the standard PWM or the alternative BaMM, which incorporates the framework of PWM, but (b) the mild or extra mild thresholds engage the better performance of both alternative models BaMM and SiteGA.

**Figure 2:**
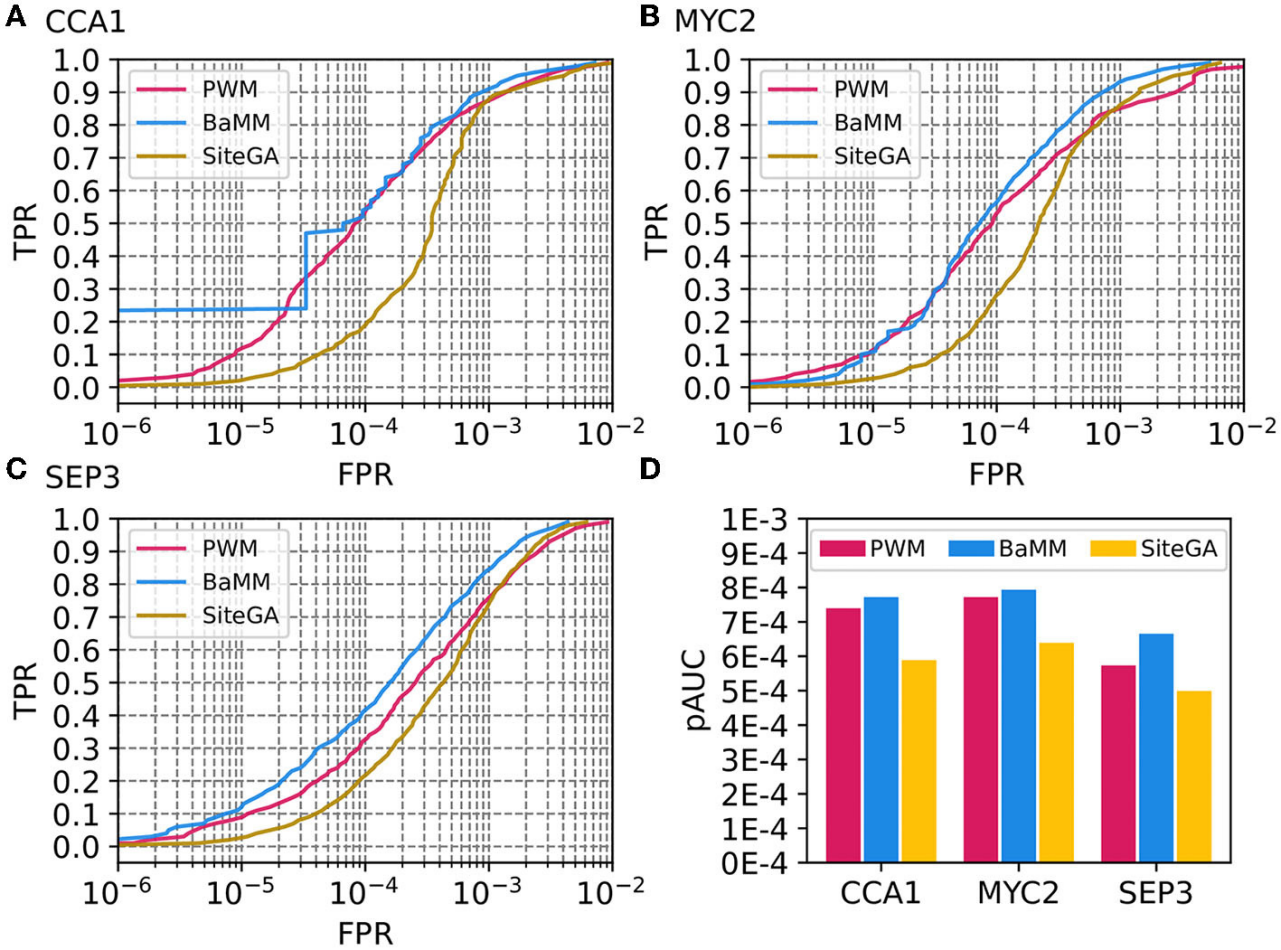
Performances of PWM, BaMM, and SiteGA motif recognition models for three example ChIP-seq datasets. **(A–C)** Display ROC curves for datasets of CCA1, MYC2, and SEP3 TFs. We applied the bootstrap cross-validation procedure (see Section 2) and computed for each TPR, (axis Y) FPR values (axes X). **(D)** Compares performance estimates pAUC for three models and three datasets.

For more detailed analysis, we selected three ChIP-seq datasets for CCA1, MYC2, and SEP3 TFs (GSM1808452, GSM3856417, and GSM1279838/GSM1279839, respectively). We ensured that predicted sites for these three ChIP-seq datasets for models PWM, BaMM, and SiteGA are significantly similar (similarity of motifs, *p*-value < 0.05) to the known motifs for the target TFs from the CIS-BP database (Weirauch et al., 2014). Figures 2A–C shows ROC curves for three datasets; Figure 2D compares the performance measure pAUC for them. Overall, for three datasets, PWM and BaMM possess almost equal accuracy superior to that of SiteGA. Despite the SiteGA model possessing the worst performance among three models in the range of stringent (FPR ≤ 1E-4) and medium (1E-4 < FPR ≤ 2.5E-4) thresholds, PWM and SiteGA models show similar performance for the mild (2.5E-4 < FPR ≤ 5E-4) and extra mild (5E-4 < FPR ≤ 1E-3) thresholds. This result is in good accordance with the behavior of the ROC curves respecting the benchmark collection (Figure 1A).

### 3.2. The motifs of PWM, BaMM, and SiteGA models show a distinct structure

**Figure 3:**
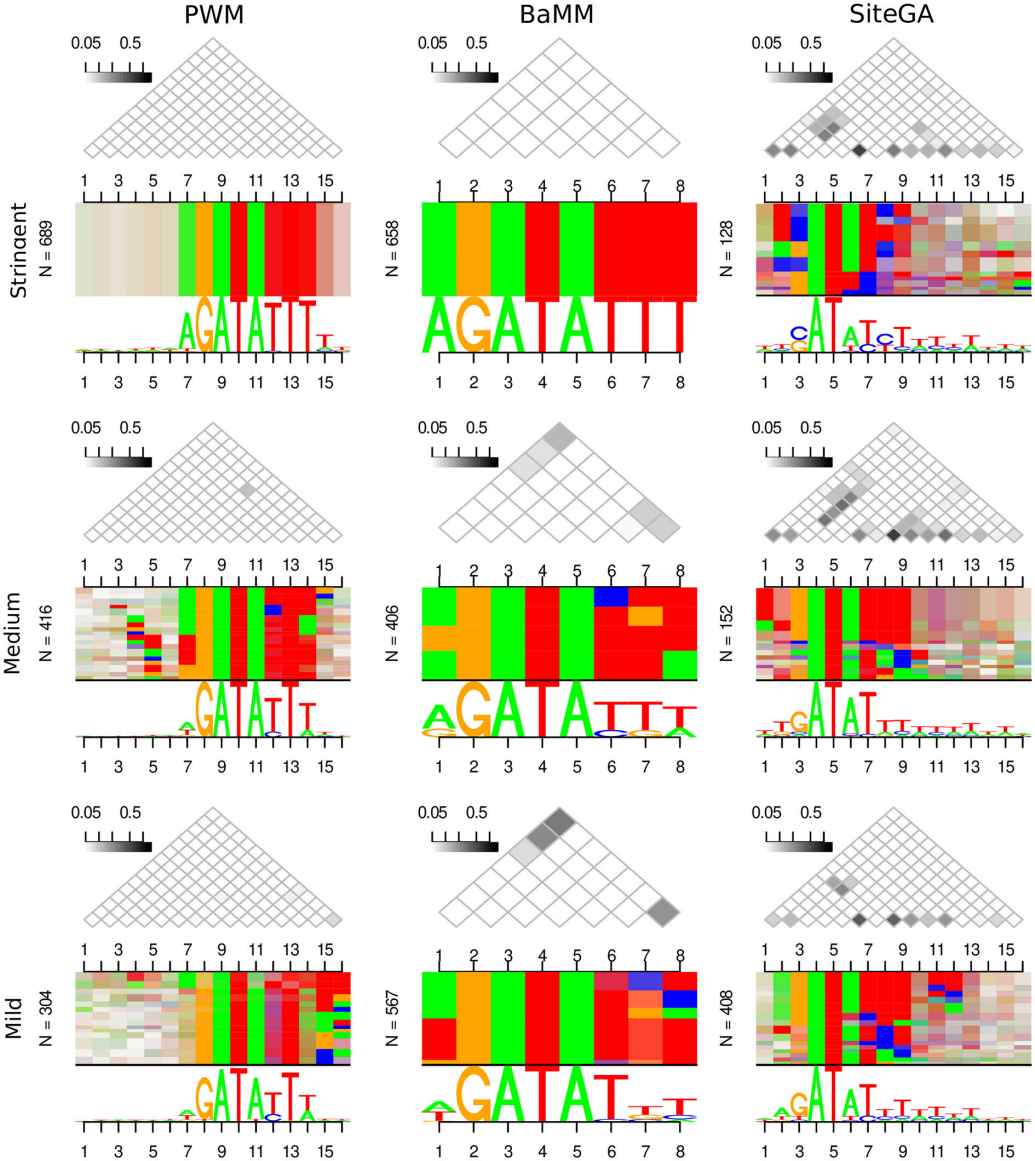
Traditional and alternative sequence logos represent the alignments of sites predicted by PWM, BaMM, and SiteGA recognition models for CCA1 TF. Three columns show three models. Three rows show the growth of the recognition score: the bottom, middle, and top rows depict the mild, medium, and stringent ranges of recognition scores (2.5E-4 < ERR ≤ 5E-4, 1E-4 < ERR ≤ 2.5E-4, and ERR ≤ 1E-4, respectively, see Section 2). In each of the 3 × 3 cells, the traditional sequence logo is located under the alternative sequence logo (DepLogo, Grau et al., 2019). Above each alternative logo, the triangle matrix shows the mutual information as a measure of the position interdependency. The dependencies are visualized as horizontal boxes showing pairs of interacting nucleotides; from the left side of each logo, the total number of BSs (N) is designated.

The computed above estimates for the recognition performance represent only how well we distinguish between the functional and non-functional sites. Hence, next, we enquired whether the different motif models were able to complement each other, i.e., they represented the sites of distinct structures. The simplest approach to answer this question is to compare the traditional sequence logos for the predicted sites of different models. To visualize these logos in detail of the estimated binding affinity, for each model, we compiled the list of predicted BSs in all peaks and sorted them in the descending order of the model's recognition score. Next, we considered the stringent, medium, and mild recognition scores ranges for ERR ≤ 1E-4, 1E-4 < ERR ≤ 2.5E-4, and 2.5E-4 < ERR ≤ 5E-4, correspondingly (see Section 2). However, the traditional sequence logo is unable to represent dependencies between various positions of predicted BSs. We performed the visualization of these dependencies with the alternative sequence logo DepLogo that was recently proposed (Grau et al., 2019). For a given alignment of sites, this approach classifies dependencies of nucleotide occurrences in various site positions in terms of the mutual information values and constructs the alternative sequence logos depicting these dependencies. The DepLogo approach partitions the alignment into the subsets sequentially, according to the strength of position dependencies (see example in Figure 3). The resulting clasterization shows these subsets, and within each subset, the co-occurrence of nucleotides are marked by colored boxes, so that multiple pairwise dependencies create a common pattern for a given input alignment of sites. Below all boxes, the traditional sequence logo explains the nucleotide occurrences for all positions; above all boxes, the matrix of mutual information values estimates the interaction strength for all pairs of site positions.

Supplementary Figure 4 presents the traditional and alternative sequence logos for PWM, BaMM, and SiteGA motif models for datasets of CCA1, MYC2, and SEP3 TFs. The PWM model for all datasets does not show any dependencies. Note for the PWM model, all triangle matrices do not contain gray cells, and for all positions, the even vertical stripes respect the nucleotide conservation of traditional logos shown underneath. On the contrary, the application of alternative BaMM and SiteGA models for all datasets reveal the dependency patterns, though it seems that the SiteGA model shows a higher number of dependencies. Thus, the SiteGA/BaMM shows gray cells for three/one datasets, and the absence of a PWM-like pattern of even vertical stripes is detected for three/one dataset(s), respectively. Since we observed distinct peculiarities of ROC curve behavior for PWM, BaMM, and SiteGA models, below we consider the traditional and alternative logos separately for predicted BSs of various estimated affinities.

Figure 3 and Supplementary Figures 5, 6 present the traditional and alternative sequence logos for PWM, BaMM, and SiteGA motif models for CCA1, MYC2, and SEP3 datasets, and for the stringent, medium, and mild ranges of the recognition score. The traditional logos confirm that at least part of the most conserved core sequence of a PWM (hereinafter, a core) is detected for all TFs, for all models, and all ranges of the recognition score. For various stringencies, PWM and, to a slightly lesser extent, BaMM tend to preserve the same nucleotides within a core. However, for these two models at the stringent threshold (ERR ≤ 1E-4), the conservation of nucleotides within a core (the bigger height of letters in a traditional logo) is supplemented with more conserved flanks. BaMM and SiteGA divide a core into the conserved and variable positions. For example, this effect is clearly observed for CCA1 for BaMM (polyT at 3'-flank) and for all TFs for SiteGA. SiteGA shows the most similar logos to those of PWM for the mild recognition scores. While the medium and stringent recognition scores show less similar patterns to those of PWM. We interpreted this phenomenon as the capacity of the SiteGA model to combine two factors: the dependencies of various positions within a motif, and the motif conservation in terms of a PWM model (see Supplementary methods, Equation (S1), the factors E(X) and D(X), respectively). Most probably, for the high scoring SiteGA BSs the factor of dependencies makes a greater contribution, than the factor of conservation. Thus, BaMM and SiteGA models show the motif conservation only in a part of the cores respecting PWM. While the sequences flanking these cores do not show notable conservation between three models for any TF. Overall, the traditional sequence logos respecting three models for the same TF are only moderately similar.

The alternative logos, in general, show the reciprocal patterns to those of the traditional logos (Figure 3; Supplementary Figures 4–6). Thus, the PWM model for any threshold is almost completely devoid of any dependencies. BaMM at the stringent threshold also does not show any dependencies, a small number of dependencies arise at the medium threshold, and their amount is moderately increased at the mild threshold. The SiteGA model revealed substantially higher various dependencies for all thresholds compared to BaMM. Notably, for the SiteGA model, the patterns of mutually dependent positions estimated through the triangle matrices of the mutual information values are moderately similar for various ranges of recognition scores. Concluding, the DepLogo visualization explains why the BaMM and especially the SiteGA model may lose the conservation in terms of the traditional sequence logo, but at the same time, these alternative models may provide complementary predictions to that of the PWM model. Moreover, additional information on the interdependencies of various positions explains why BaMM and SiteGA models successively compete with PWM in recognition performance, especially in the range of FPR respecting mild and extra mild recognition thresholds (2.5E-4 < FPR <1E-3, Figure 1A). The massive analysis of the benchmark collection confirmed that the PWM detects only a limited number of position dependencies, the BaMM shows a substantially higher number of dependencies, and the SiteGA model detects the dependencies almost for all datasets; the median values for the benchmark collection are 0, 2, and 11 for PWM, BaMM, and SiteGA, respectively (see Supplementary Table 3).

Next, we studied the pairwise similarity of the motif models in terms of their recognition scores. We considered three possible combinations of the models, BaMM/PWM, SiteGA/PWM, and SiteGA/BaMM. We converted the recognition scores of each model to the scale of ERR (see Section 2). For each combination of two models, we compiled the list of hits supported by these two models; the term “hit” implies the overlapping of BSs predicted by two distinct models. Supplementary Figure 7 shows the heatmaps for the abundances of the hits possessing the recognition scores for various pairwise combinations of the models. Clearly, the combination BaMM/PWM showed a better match compared to those of SiteGA/PWM and SiteGA/BaMM combinations. But even for the PWM/BaMM case, for CCA1 and MYC2, the major portion of the hits lies outside the diagonal line, i.e., a certain recognition score of the PWM model respects the greater or lesser one of the BaMM. Moreover, the combinations SiteGA/PWM, and SiteGA/BaMM showed matches of moderate quality. Concluding, the pairwise comparisons of recognition scores of PWM, BaMM, and SiteGA models suggest that these models provide only moderately similar scoring of BSs.

### 3.3. Combined application of PWM, BaMM, and SiteGA models

We have demonstrated above that the motifs predicted by PWM, BaMM, and SiteGA models possess a distinct structure in terms of traditional and alternative logos, we can expect that the fractions of recognized peaks for various models are overlapped only partially. In this section, we test whether the combined application of three models respects higher portions of recognized peaks, compared to the application of individual models.

**Figure 4:**
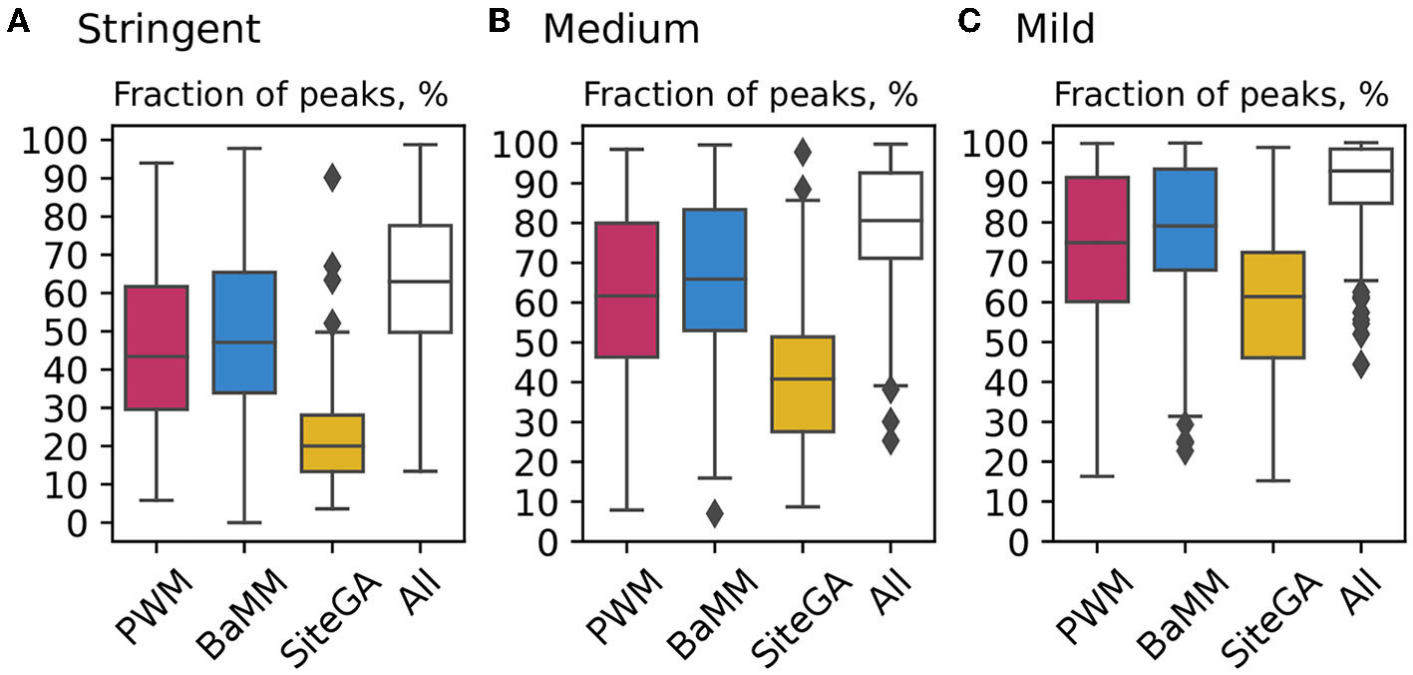
Comparison of application of PWM, BaMM, and SiteGA motif models and their combination for the benchmark collection of ChIP-seq datasets. **(A–C)** Show boxplots computed with the stringent, medium, and mild thresholds (recognition scores respecting ERR ≤ 1E-4, ERR ≤ 2.5E-4, and ERR ≤ 5E-4). Each boxplot shows the distribution of the fractions of peaks containing the BSs predicted by individual models, and the fraction of peaks containing the BSs predicted by at least one model out of three (white boxes “All”). Red, blue, and yellow columns mark PWM, BaMM, and SiteGA models, respectively. The boxplot presents the distributions of the *Q*_1_, *Q*_2_, and *Q*_3_ quartiles of the fractions of the peaks with recognized TFBS. Whiskers below/above the *Q*_1_/*Q*_3_ respect the minimum/maximum values if they were located within 1.5 interquartile ranges (*IQR* = *Q*_3_−*Q*_1_) from *Q*_1_/*Q*_3_, otherwise they are equal to {*Q*_1_−1.5*IQR}/{*Q*_3_+1.5*IQR}, respectively. In the latter case, we marked all other points as outliers.

Figure 4 for the benchmark collection of ChIP-seq datasets for stringent, medium, and mild recognition thresholds compare the distributions of fractions of the peaks with TFBS predicted by individual models with those computed by the combined application of three models. It suggests that at the stringent threshold (a) the fractions of peaks with PWM and BaMM BSs are very similar, (b) the fraction of peaks with SiteGA BSs is more than twice less (the median values for PWM, BaMM and SiteGA are 43.4%, 47.1%, and 20%, respectively). However, compared to PWM/BaMM, the SiteGA model shows the most notable growth in the fraction of peaks in the transition from the stringent to the medium threshold (medians rise to 61.7, 65.9, and 40.8%) and from the medium to the mild threshold (74.9, 79.1, and 61.4%). This means that (a) the majority of PWM hits possess “high conservation,” while the majority of SiteGA hits show “low conservation”; (b) BaMM BSs most probably are ambiguous; for the stringent/medium thresholds, they are similar to those of PWM; while for the mild threshold, BaMM predicts distinct ‘low conservation’ hits associated with the dependencies of positions.

Regardless of the threshold selection, the combined application of three models adds about 15% to a model with the maximal fraction of recognized peaks (15.9, 14.7, and 13.8% for the stringent, medium and mild thresholds, respectively, Figure 4). Next, we considered the background datasets, i.e., those that are opposite to the foreground datasets (ChIP-seq peaks) in the training of models (see Section 2, Supplementary methods). The analysis of the respective collection of the background datasets (Supplementary Figure 8) confirmed that the fractions of individual models and those for the combined application of three models were substantially less than those for the foreground datasets.

**Figure 5:**
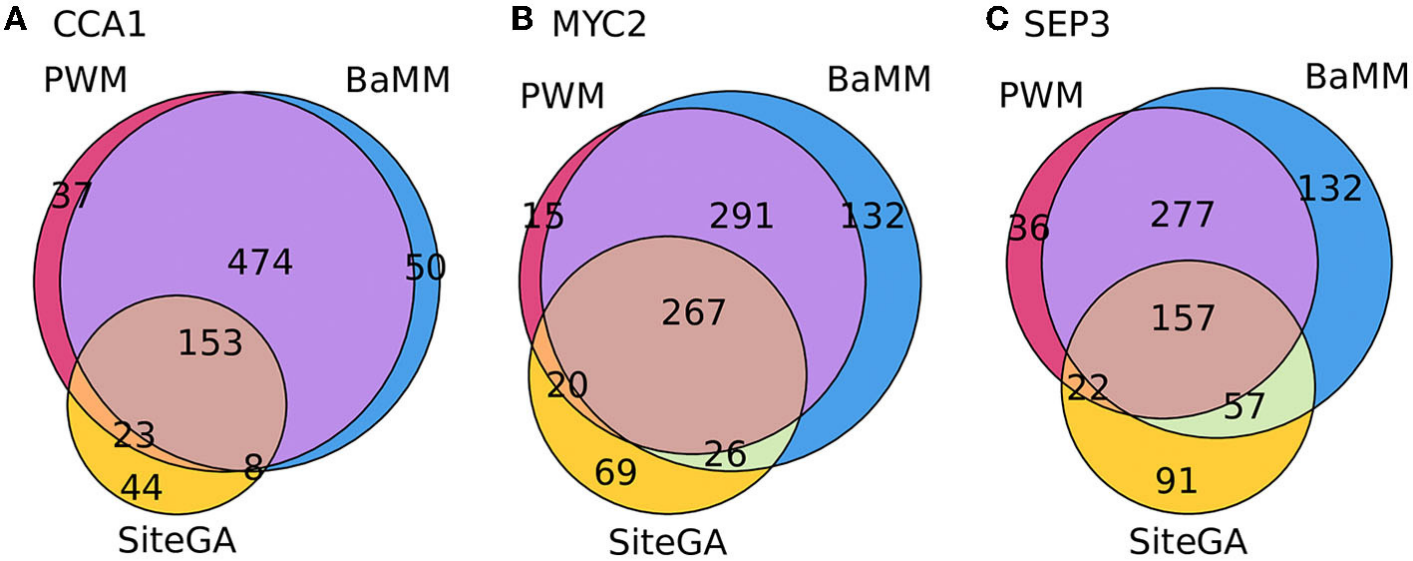
Venn diagrams for overlaps between the fractions of peaks containing the BSs predicted by three different models. **(A–C)** Show CCA1, MYC2, and SEP3 datasets, respectively. Numbers denote the number of peaks. Totally, each dataset consists of 1,000 peaks. The analysis was performed with the medium thresholds (recognition scores respecting ERR ≤ 2.5E-4).

Figure 5 depicts the Venn diagrams for the classification of peaks predicted by only individual models, and certain combinations of two, or three models. It can be concluded that (a) the overwhelming majority of the peaks containing either hits of PWM or BaMM models possessed hits of both models; (b) for pairwise combinations, PWM/SiteGA and BaMM/SiteGA, i.e., if we considered the fractions of peaks containing the hits of SiteGA, then about a third of these fractions respected prediction of the sole SiteGA model. Concluding, the combined application of standard and alternative models extended the portions of predicted peaks compared to those predicted by any single model. Alternative motif models BaMM and SiteGA detected the prominent portions of the peaks that were not detected by the standard PWM.

**Figure 6:**
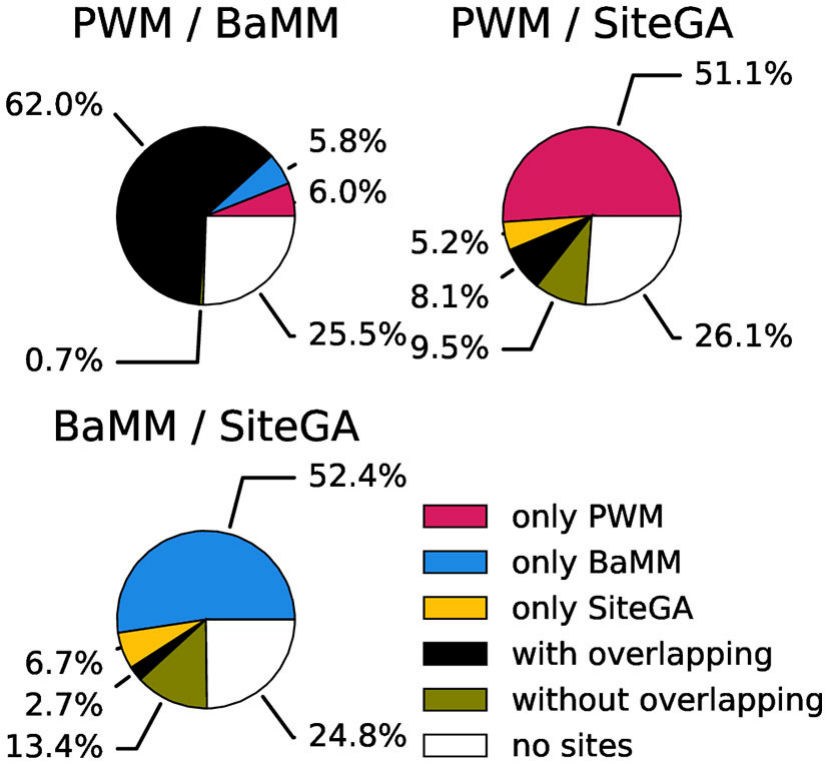
Classification of peaks from the CCA1 dataset taking into account the presence of the BSs and the overlaps of their positions. Three pie charts show pairwise combinations of PWM/BaMM, PWM/SiteGA, and BaMM/SiteGA models. Red, blue, and yellow sectors mark the fractions of peaks recognized by only one model in pairs (PWM, BaMM, and SiteGA, respectively). Black/olive sectors denote the fractions of peaks recognized by two models with/without overlapping hits. The white color means that the hits of both models are absent. The analysis was performed with the medium thresholds (recognition scores respecting ERR ≤ 2.5E-4).

Next, since the large fractions of peaks contained the hits of at least two models, we estimated how often the presence of hits of two distinct models in a peak implied the overlapping of their positions. We studied three pairwise combinations of models PWM/BaMM, PWM/SiteGA, and BaMM/SiteGA. We measured the fractions of peaks with the hits predicted by individual models and divided the fractions of peaks with the hits predicted by two models on two subfractions with present/absent overlapping of the hits positions. Figure 6; Supplementary Figure 9 show the results of this classification for ChIP-seq datasets for CCA1, MYC2, and SEP3. Obviously, in the case of PWM/BaMM, the presence of the hits of these models in a peak almost always meant an overlapping of the hits. On the contrary, in the cases of PWM/SiteGA and BaMM/SiteGA, the fractions containing the hits predicted by two models were almost equally divided between overlapped and non-overlapped subfractions.

### 3.4. GO analysis

To perform GO enrichment analysis for predicted TFBS hits of three recognition models, we mapped the peaks to *A. thaliana* genes and predicted within the peaks BSs (see Section 2). Thereafter, we detected the lists of GO terms for biological processes enriched in the gene lists for all peaks, and for their TFBS predicted by PWM, BaMM, or SiteGA models (see Supplementary Tables 4–6). In particular, for each GO term, we computed the significance of enrichment (adjusted *p*-value, p_adj) and the fold enrichment (see Table 1 and Section 2).

Supplementary Figures 10–12 display the significantly enriched GO terms for CCA1, MYC2, and SEP3 datasets. Among the top-ranked terms for the CCA dataset for three models, we found the terms “circadian rhythm,” “rhythmic process,” and “response to cold.” Concerning MYC2, about half of the top-ranked GO terms correspond to all peaks and three models (e.g., “response to wounding,” “response to jasmonic acid,” and “response to fatty acid”), but almost all the rest half is detected only for the SiteGA model. The top-ranked GO terms for SEP3 “flower development,” “floral organ development” also respect all peaks and three models. Some less significant GO terms show the significance only for one model. For CCA1, MYC2, and SEP3 datasets 1/16/0, 2/7/16, and 13/20/15 terms were specifically detected for PWM/BaMM/SiteGA outputs, respectively (Supplementary Tables 4–6). This proposes that BSs of the alternative models correspond to specific biological functions of genes. Notably, earlier we already observed the specificity of predictions of the SiteGA model for ChIP-seq data of human SF-1 TF, these BSs had a visible trend to GO terms related to negative regulation and apoptosis (Levitsky et al., 2016).

**Figure 7:**
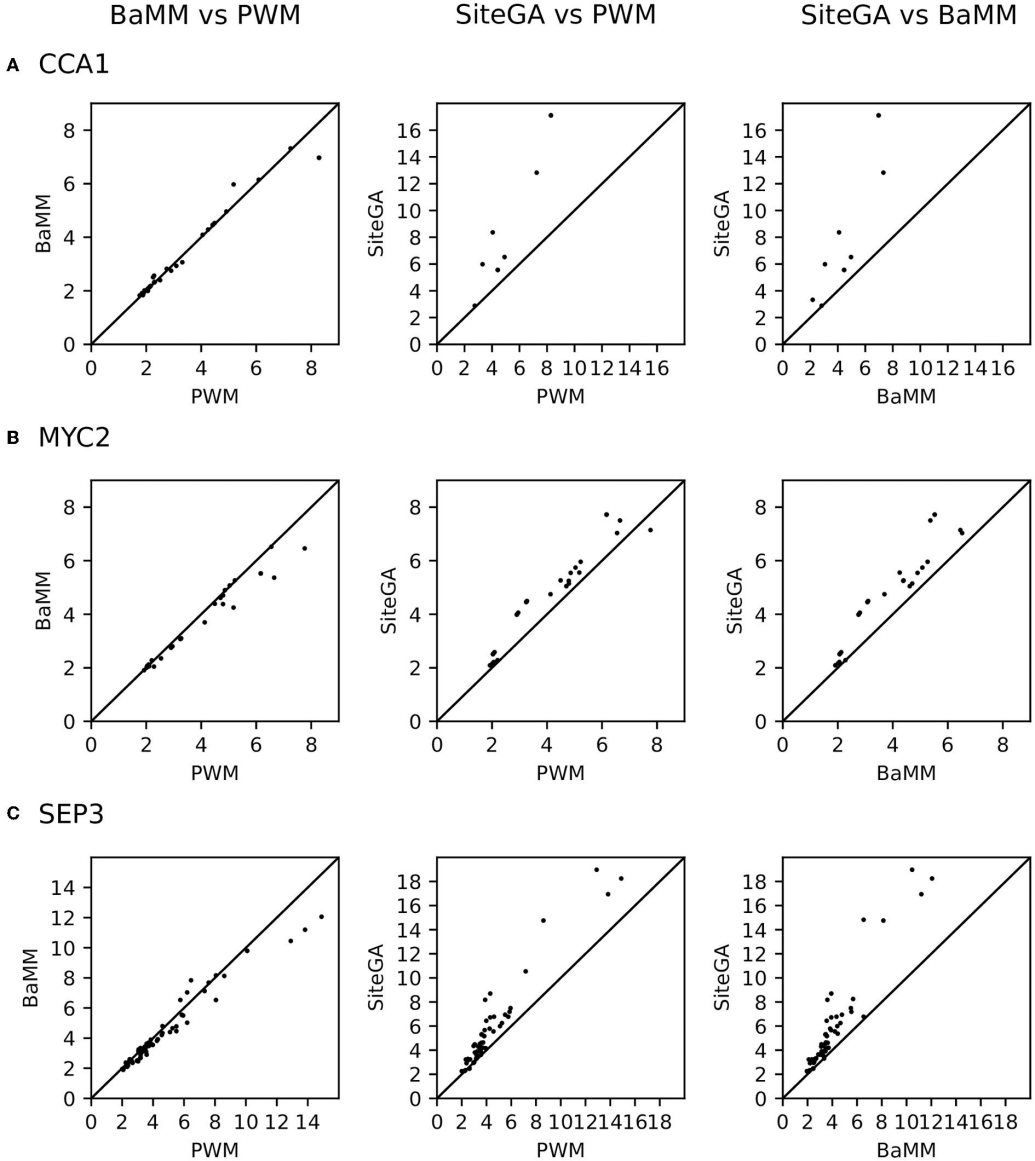
Efficiency of PWM, BaMM, and SiteGA models in terms of the portions of genes with the significantly enriched GO terms among all genes with predicted sites. **(A–C)** Show CCA1, MYC2, and SEP3 datasets, respectively. Axes in all scatterplots imply the fold enrichment respecting the predictions of models (see Table 1 and Section 2). For each dataset, we included in analysis the same set of the GO terms; each GO term possessed the significance of enrichment (*p*_adj < 0.05) for three motif models simultaneously.

**Figure 8:**
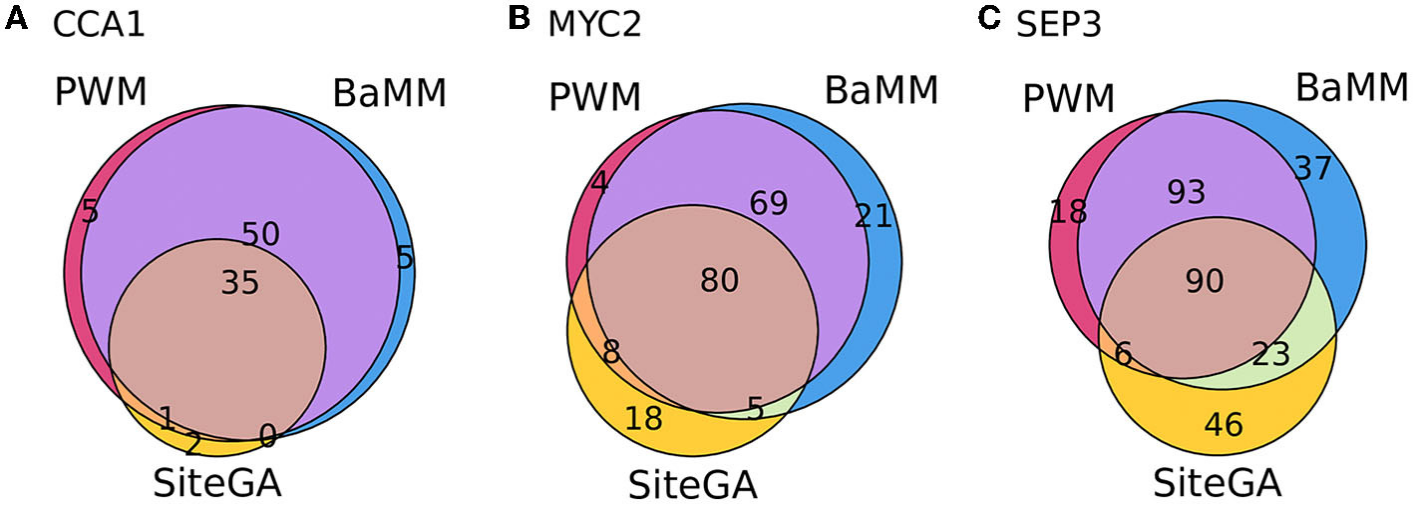
Venn diagrams for overlaps between the number of genes containing the sites predicted by PWM, BaMM, and SiteGA motif models and supported by the significantly enriched GO terms. **(A–C)** Show CCA1, MYC2, and SEP3 datasets. For each dataset, we included in the analysis the same set of the GO terms; each GO term possessed the significance of enrichment (*p*_adj < 0.05) for three motif models simultaneously.

We found that for the overwhelming majority of the significant GO terms (*p*_adj < 0.05, Supplementary Tables 4–6), the SiteGA model possesses higher fold enrichments compared to those of other models. For example, for CCA1, MYC2, and SEP3 datasets, the corresponding first ranking terms “circadian rhythm,” “response to wounding,” and “flower development” show for the PWM/BaMM/SiteGA models the fold enrichments 4.42/4.46/5.56, 4.79/4.38/5.25, and 3.60/3.44/5.29, respectively. We included in the analysis all GO terms with significant enrichment for any of three motif models; this yielded 9, 27, and 58 GO terms for CCA1, MYC2, and SEP3 datasets, respectively (*p*_adj < 0.05, see Supplementary Tables 4–6). The scatterplots in Figure 7 depict the pairwise comparisons of the models BaMM/PWM, SiteGA/PWM, and SiteGA/BaMM in the fold enrichments for these commonly detected significant GO terms. In the pairs of models, SiteGA/PWM and SiteGA/BaMM, the SiteGA model has the higher/lower fold enrichments in 9/1 and 10/0 GO terms for CCA1, 26/1 and 27/0 for MYC 2, 57/3 and 60/1 for SEP3. A ratio between the fold enrichments of two models (Figure 7) estimates the efficiency of their application for the prediction of the genes with a specific GO term since for these two models the frequency of all genes annotated with this GO term is the same (see Table 1). The higher efficiency of the SiteGA model compared to those of other motif models may reflect both (a) more accurate predictions in the genes that are also predicted by PWM/BaMM models and (b) the extension of the SiteGA predictions to the genes lacking the predicted sites of other models. Separately for each dataset and for three motif models, we considered all genes and all significant GO terms (*p*_adj < 0.05, see Supplementary Tables 4–6). The classification of these genes (Figure 8) suggests that both alternative motif models substantially extend the predictions of the traditional PWM model in the genes supported by significantly enriched GO terms.

Overall, GO analysis confirms that the combined application of traditional and alternative models considerably extend the prediction of BSs in the genes possessing the significantly enriched annotations of the biological processes.

## 4. Discussion

Gene expression regulation is the main function of TFs carried out through their binding to genomic DNA. One of the important tasks of modern molecular biology is to identify these binding sites, TFBSs. ChIP-seq is a widely applied experimental technique to deduce the genome-wide sequence specificity of TF binding in various cell/tissue types. The popular approach to find out the genome-wide TFBS nucleotide context specificity are as follows: (a) to use ChIP-seq peaks for consequent *de novo* motif search and (b) to model in this search TFBS by a PWM (Lloyd and Bao, 2019; Ma et al., 2019). This approach verifies the reliable motifs of the target TFs for only a part of ChIP-seq peaks (Hunt et al., 2014). We propose that the rationale to this discrepancy comes from the structural heterogeneity of TFBSs (Kim et al., 1995; Merkulov and Merkulova, 2009; Omelina et al., 2011; Mitra et al., 2018; Morgunova et al., 2018; Chen et al., 2019; Rogers et al., 2019). Earlier we proved that for two mammalian TFs, FOXA2 and SF1, combinations of several recognition models based on different principles allowed to increase the portion of peaks containing predicted sites compared to the application of the sole PWM models (Levitsky et al., 2014, 2016). In particular, the integration of PWM/diPWM (ChIPMunk/diChIPMunk) models (Kulakovskiy et al., 2010, 2013) and the SiteGA model (Levitsky et al., 2007) allowed the identification of FOXA2 sites in up to 90% of the ChIP-seq loci (Levitsky et al., 2014). Notably, there was no literature data on the indirect interaction of FOXA2 TF with DNA.

Recently, we developed the MultiDeNA approach to combine and uniformly apply distinct *de novo* motif models for ChIP-seq data analysis (Tsukanov et al., 2021). We approved this approach for 22 ChIP-seq datasets of mammalian TF FOXA2 and considered PWM, diPWM, BaMM, and InMode TFBS models. On average, we confirmed potential TFBS in about 74% of peaks, while the sole PWM model verified only 47% of them. We believe that a combination of several methodologically distinct motif models to deduce TFBS nucleotide context specificity from ChIP-seq data may extend the fraction of verified ChIP-seq peaks. This extension is a consequence of various mechanisms of TF-DNA interaction, e.g., including cooperative binding with other TFs (Slattery et al., 2014; Morgunova and Taipale, 2017).

In the current study, we compiled the benchmark collection of 111 ChIP-seq datasets for TFs from *A. thaliana* (Supplementary Table 1). We approved the reliability of this collection with known motifs for target TFs, a few datasets with missing enrichment of target TF's motifs may be explained either by the tethering (indirect DNA binding; Yu et al., 2021), co-binding with partner TFs (Levitsky et al., 2020; Yu et al., 2021) since we took into analysis the first ranking motif from each model or available experimental data (in most cases *in vitro*) on particular TFs still were not complete to describe *in vivo* DNA affinity (see column “Motif Source” in Supplementary Table 1).

We applied three methodologically different motif models to plant ChIP-seq data. The first model is PWM in the STREME implementation (Bailey, 2021). PWM is the most popular and conventional TFBS model used in ChIP-seq data analysis. The second model BaMM applies the framework of PWM, i.e., independent contributions from site positions to the total binding affinity, and extends them with impacts of dependencies between close positions (Siebert and Söding, 2016). The third model SiteGA is developed independently from the PWM, it deduces the set of most important locally positioned dinucleotides with mutual dependencies within potential sites (Levitsky et al., 2007; see also Supplementary methods).

Comparisons of the ROCs curves for PWM, BaMM, and SiteGA models computed for the benchmark collection of 111 ChIP-seq datasets (Figure 1A), and those for three example datasets (Figures 2A–C) suggest that PWM and BaMM show a better performance for the stringent and medium thresholds, while BaMM and SiteGA show a better performance for the mild and extra mild thresholds. We may interpret this as follows. The BaMM framework incorporates the standard PWM model. If for the stringent and medium thresholds the hypothesis of position independence is justified, then (a) the impacts to the total recognition score of BaMM from the dependent positions are too small compared to those from independent positions, i.e., BaMM and PWM provide similar results and (b) the recognition performance of the SiteGA model is bad since this model does not use the framework of the PWM model directly as BaMM does. With regard to the mild and extra mild thresholds, the PWM model compiles less frequent nucleotides even within the core consensus; the transition to milder thresholds most probably destroys the occurrence of nucleotides within the most conserved positions of a core consensus. Hence, a negative influence on the total recognition score of these minor nucleotides within the core consensus probably is better compensated through multiple dependencies between positions. These dependencies are involved in alternative BaMM/SiteGA models, but they are totally absent in the standard PWM. Consequently, for the mild and extra mild thresholds, the PWM allows changing nucleotides in a certain position according only to respective weights in a column of matrix (i.e., independently from other positions), while this mutation process for the BaMM/SiteGA models are restricted by certain dependencies of this position with other positions.

Our results on visualization of TFBS with estimated high, medium, and low affinities with the traditional and alternative logos indicate that PWM, BaMM, and SiteGA models provide distinct structures of TFBS. While the popular PWM model reveals only the context pattern of the canonical consensus, and, respectively, the predicted BSs show strict consensuses at the stringent threshold, and this pattern is gradually blurred at the mild threshold (Figure 3; Supplementary Figures 5, 6). The dependency logos for the PWM models for the ChIP-seq datasets of CCA1, MYC2, and SEP3 TFs are almost completely devoid of the dependencies between the occurrences of nucleotides in different positions (Figure 3; Supplementary Figures 4–6). The BaMM shows a moderate number of dependencies, and the SiteGA model shows the highest results (BaMM exceeds PWM, and SiteGA exceeds BaMM for about 70 and 95% of all the datasets, see Supplementary Table 3). For any threshold, and for all three TFs, the SiteGA model shows the mosaic pattern in the dependency logo. This pattern reflects a number of various dependencies between the nucleotide occurrences in different positions. Thus, the visualization of BSs predicted by the PWM, BaMM, and SiteGA models with the traditional sequence logo and the dependency logo suggests that these models provide mutually complementary results. While the canonical PWM reveals the canonical consensus but ignores possible interaction of positions; the alternative BaMM model extends the traditional model and uses its framework to incorporate the dependencies of positions. The SiteGA model preserves only the main frame of the traditional consensus, but this model deduces the integrated pattern of mutually dependent dependencies of locally positioned nucleotide contexts within a predicted motif. The dependency logo supports the considerable advance of alternative BaMM and SiteGA models in explaining the “non-canonical” TF binding *in vivo*. The results of the analysis of predicted BSs with the traditional sequence logo and the dependency logo are in good accordance with many recent studies. The analysis of *A. thaliana* MYC2 *in vivo* and *in vitro* genomic binding data (López-Vidriero et al., 2021) confirmed that a particular nucleotide composition of flanking sequences for MYC2 binding sites is necessary for MYC2 binding. Another study (Käppel et al., 2021) found several diverse core binding sites of *A. thaliana* SEP3 (SEPALLATA3) TF *in vitro*. However, the different cores could act as SEP3 binding sites, preferred AT-rich flanking motifs were almost always the same.

Pairwise comparison of recognition scores of PWM, BaMM, and SiteGA models revealed the moderately similar estimated affinity for PWM/BaMM, while the scores for PWM/SiteGA and BaMM/SiteGA pairs show more diverse estimated affinities (Supplementary Figure 7). We propose the following explanation for these results. The BaMM methodologically extends PWM, while the SiteGA is an independent model from PWM.

Next, we enquired whether PWM, BaMM, and SiteGA models provide almost complete verification of TFBS in peaks. A comparison of the application of individual models and their combinations for the benchmark collection of ChIP-seq datasets confirmed that almost half of ChIP-seq data contain the conserved consensuses (Figure 4). For the stringent threshold, the fraction with recognized SiteGA BSs is too small compared to those of PWM/BaMM (20 vs. 43.4/47.1%). The transition from the stringent to the medium threshold shows that the additions of all the models are similar (the median increases to 18.3, 18.8, and 20.8% for PWM, BaMM, and SiteGA, respectively). Note that SiteGA shows the greatest increment, and BaMM slightly exceeds PWM. The transition from the medium to the mild threshold indicates that the additions of SiteGA are notably larger than those for other models (13.2, 13.2, and 20.6% for PWM, BaMM, and SiteGA, respectively). Hence, PWM representation (i.e., conventional conserved consensus) is more accurate for the stringent and medium thresholds, while BaMM/SiteGA models that take into account the position dependencies provide the greater additions for the mild threshold. Notably, the model SiteGA that takes into account arbitrary dependencies shows the greatest addition for the transition from the medium to the mild threshold. To discuss the analysis of the Venn diagrams, we consider the fractions of the peaks containing the predictions of an individual model among all the peaks predicted by this model. We may conclude that for all three tested TFs (Figure 5), these fractions are notably smaller for PWM and BaMM than those for the SiteGA model. Hence, the SiteGA model is able to recognize potential sites of diverse structures compared to those of PWM and BaMM. This conclusion is additionally supported by the classification of the peaks of three datasets taking into account the presence of BSs and the overlaps of their positions (Figure 6; Supplementary Figure 9).

Finally, the GO enrichment analysis for CCA1, MYC2, and SEP3 datasets (Supplementary Figures 10–12; Supplementary Tables 4–6) confirms the consistency of the previous conclusions. Though the most significant GO terms are the same for PWM, BaMM, and SiteGA models, the alternative BaMM/SiteGA models predict sites of the diverse structure relative to those predicted by PWM since these models possess additional specific significant GO terms. Finally, we performed the analysis of the fold enrichments, respecting the significantly enriched GO terms commonly detected by all models (see Section 2; Table 1; Supplementary Tables 4–6). SiteGA systematically demonstrated the higher fold enrichments compared to those of PWM and BaMM (Figure 7). Hence, among the three motif models, the SiteGA had the highest ratios between the number of predicted genes possessing the significantly enriched GO terms and the total number of predicted genes. The classification of these genes for the PWM, BaMM, and SiteGA models (Figure 8) suggests that the higher fold enrichment of the SiteGA model is the result of extending its predictions to genes without the predictions of other models. The extensions in the number of genes respecting two alternative models BaMM/SiteGA, compared to that respecting PWM, are relatively small for the CCA1 dataset (5 and 5/2 genes are specific for individual models PWM and BaMM/SiteGA, Figure 8A), but these extensions are distinctly seen for MYC2 and SEP3 datasets (Figures 8B,C). This conclusion is confirmed by the respective analysis of peaks (for CCA1 only 37 and 50/44 peaks are specific for individual models, Figure 5). Possibly, the higher abundance of the predicted non-canonical BSs for MYC2/SEP3 TFs compared to that for CCA1 TF, is explained by the specific biological functions of these TFs. Thus, the first ranking significant GO terms “circadian rhythm,” “response to wounding,” and “flower development” that are detected, respectively, for CCA1, MYC2, and SEP3 datasets (Supplementary Tables 4–6) argue that the CCA1 TF is directly related to the housekeeping functions, while MYC2/SEP2 TFs are more prone to the condition/tissue specific functions.

We considered the benchmark collection of 111 ChIP-seq datasets for *A. thaliana* TFs. The analysis of enrichment of the known motifs for target TFs confirmed the consistency of this collection. To recognize motifs of target TFs, we applied three methodologically distinct motif models: the traditional PWM neglecting dependencies between nucleotide occurrences in various positions, and its alternatives BaMM, and SiteGA permitting dependencies within the close and arbitrary positions, respectively. Noteworthy, the BaMM extended the PWM approach, while SiteGA was developed independently from the PWM approach. We performed the bootstrap cross-validation procedure that selected the parameters of models and compared their recognition performance. We assessed that PWM and BaMM models showed the superior performance for the stringent and medium threshold, while SiteGA and BaMM models reached the superior performance for the mild and extra mild thresholds. We selected for further detailed analysis three ChIP-seq datasets for CCA1, MYC2, and SEP3 TFs. The visualization of BSs respecting various predicted affinities for three models with the traditional logos suggested that the transition from stringent to mild thresholds led to the gradual destruction of a PWM consensus approximately equal in all positions, while the alternative BaMM and SiteGA models distinguished more and less conserved positions within a consensus. We believe that these less conserved positions reflect their dependencies with other positions. The visualization by the dependency logo provided direct evidence that BaMM and SiteGA models deduced the specific patterns of mutually dependent nucleotide occurrences almost completely invisible with a traditional sequence logo. Notably, the dependency logo also proved that such dependencies were substantially weaker or they were found at a much lower frequency in potential BSs of the PWMs. The analysis of the changes in the fractions of peaks with predicted BSs depending on the recognition threshold for the whole benchmark collection proposed that PWM BSs were clearly detected already for the stringent and medium thresholds; the transition from them to milder ones showed greater impacts of alternative models compared to those of PWMs. Hence, the traditional PWM model deduces a canonical motif of high affinity for a target TF. More advanced BaMM/SiteGA models further specify this motif, and this specification is clearer for BSs of low affinity. Finally, GO enrichment analysis confirmed that alternative models may notably extend predictions of PWM in the genes possessing the inherent significant GO terms of the target TFs. For CCA1 and MYC2/SEP3 TFs, which have predominant housekeeping and condition/tissue specific biological functions, we found a lower and higher abundance of predicted binding sites specific for alternative motif models, both in peaks and in genes supported by the significantly enriched GO terms. Overall, the combined application of the traditional PWM and the alternative methodologically diverse BaMM and SiteGA models respect various TF-DNA interaction options *in vivo*, therefore, our approach notably extends a pool of context-specific TFBS in ChIP-seq data.

## Data availability statement

Publicly available datasets were analyzed in this study. This data can be found at: https://gtrd.biouml.org.

## Author contributions

AT developed the implemented algorithms, analyzed data, performed the statistical analysis, and drafted the manuscript. VM developed the study and contributed to the writing of the manuscript. VL developed the implemented algorithms, analyzed and interpreted data, developed the study, supervised the project, and drafted and wrote the manuscript. All authors contributed to the article and approved the submitted version.

## Funding

The development and implementation of motif search tools and statistical analysis were supported by Russian Science Foundation Project 21-14-00240.

## Conflict of interest

The authors declare that the research was conducted in the absence of any commercial or financial relationships that could be construed as a potential conflict of interest.

## Publisher's note

All claims expressed in this article are solely those of the authors and do not necessarily represent those of their affiliated organizations, or those of the publisher, the editors and the reviewers. Any product that may be evaluated in this article, or claim that may be made by its manufacturer, is not guaranteed or endorsed by the publisher.
