# Supplementary material for: Motif models proposing independent and interdependent impacts of nucleotides are related to high and low affinity transcription factor binding sites in Arabidopsis
Source: Front Plant Sci. 2022 Jul 28;13:938545. doi: 10.3389/fpls.2022.938545 (PMC9373801; doi:10.3389/fpls.2022.938545)
Supplement: Supplementary file 1 [file Data_Sheet_1.PDF]

Supplementary Material

1 SUPPLEMENTARY FIGURES

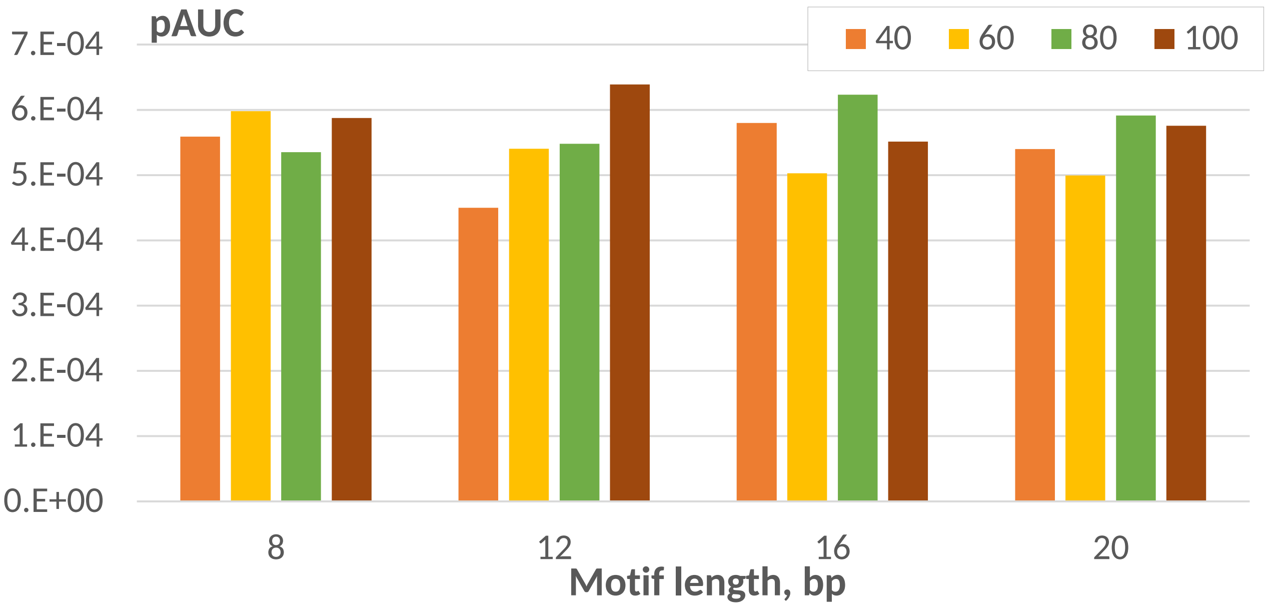

Figure S1. The selection of two parameters (the motif length, the number of LPDs) for the SiteGA model for the ChIP-seq dataset for MYC2 TF. Axis X denotes the motif length, for each one various colors mark models with various numbers of LPDs. Axis Y shows the performance measure pAUC. The maximal pAUC value 0.00063901 respects the motif length of 12 bp and the number of LPDs equal to 100. As a result, we trained the SiteGA model with the motif length of 12 bp and the number of LPDs equal to 100.

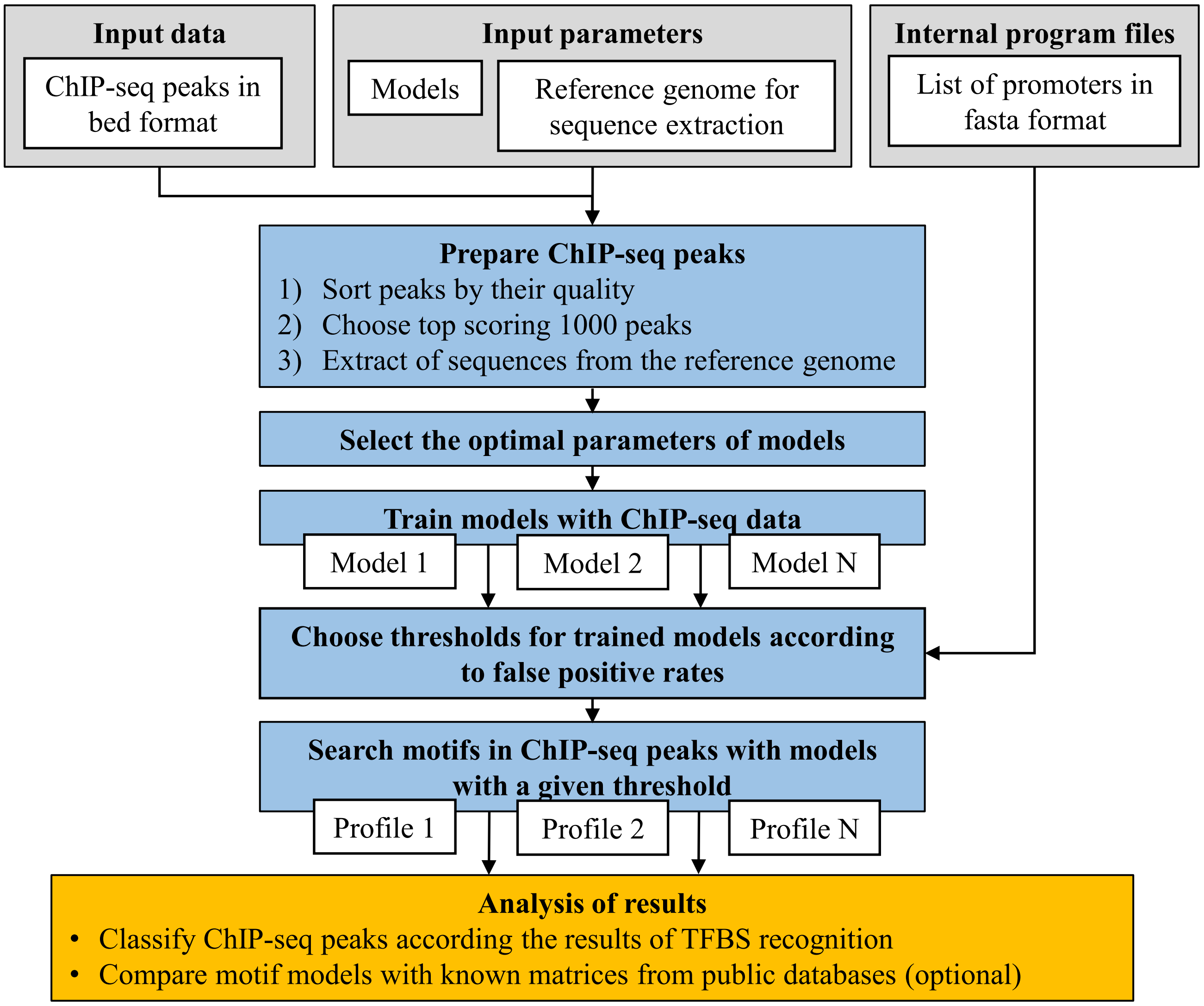

Figure S2. The scheme of the MultiDeNA workflow.

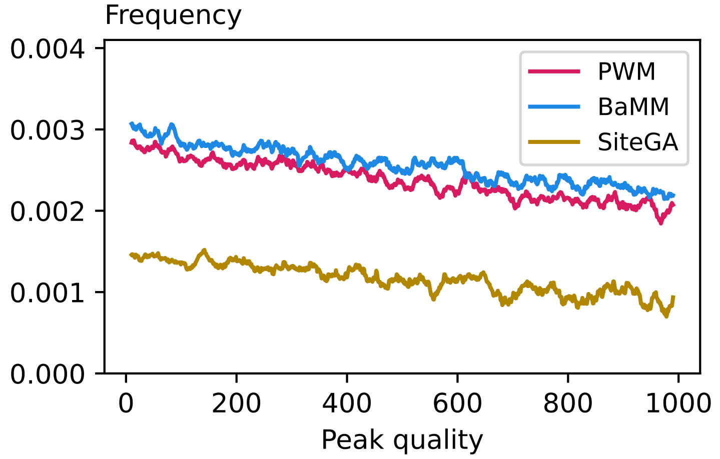

Figure S3. The distribution of the frequency of predicted BSs by the PWM, BaMM and SiteGA motif models according to the peak quality. The preparation of the raw data (Kolmykov et al., 2021) included the sorting of peaks according to the value $-10*\log_{10}$(p-value) that characterized the peak quality. This value was previously calculated for each peak by the MACS2 program (Zhang et al., 2008). Axis X shows the descending order of the peak quality; the range of quality ranks from 1 to 1000 respects the whole ChIP-seq dataset. Axis Y implies the moving average (window of 10 peaks) for the medians of the frequency of predicted BS with different quality ranks. The medians were computed for each peak quality for the benchmark collection of 111 A. thaliana ChIP-seq datasets. The frequency means the ratio of the number of hits to the number of possible positions for them in a peak. We performed calculations for the mild motif recognition threshold (recognition scores respecting ERR $\leqslant$ 5E-4).

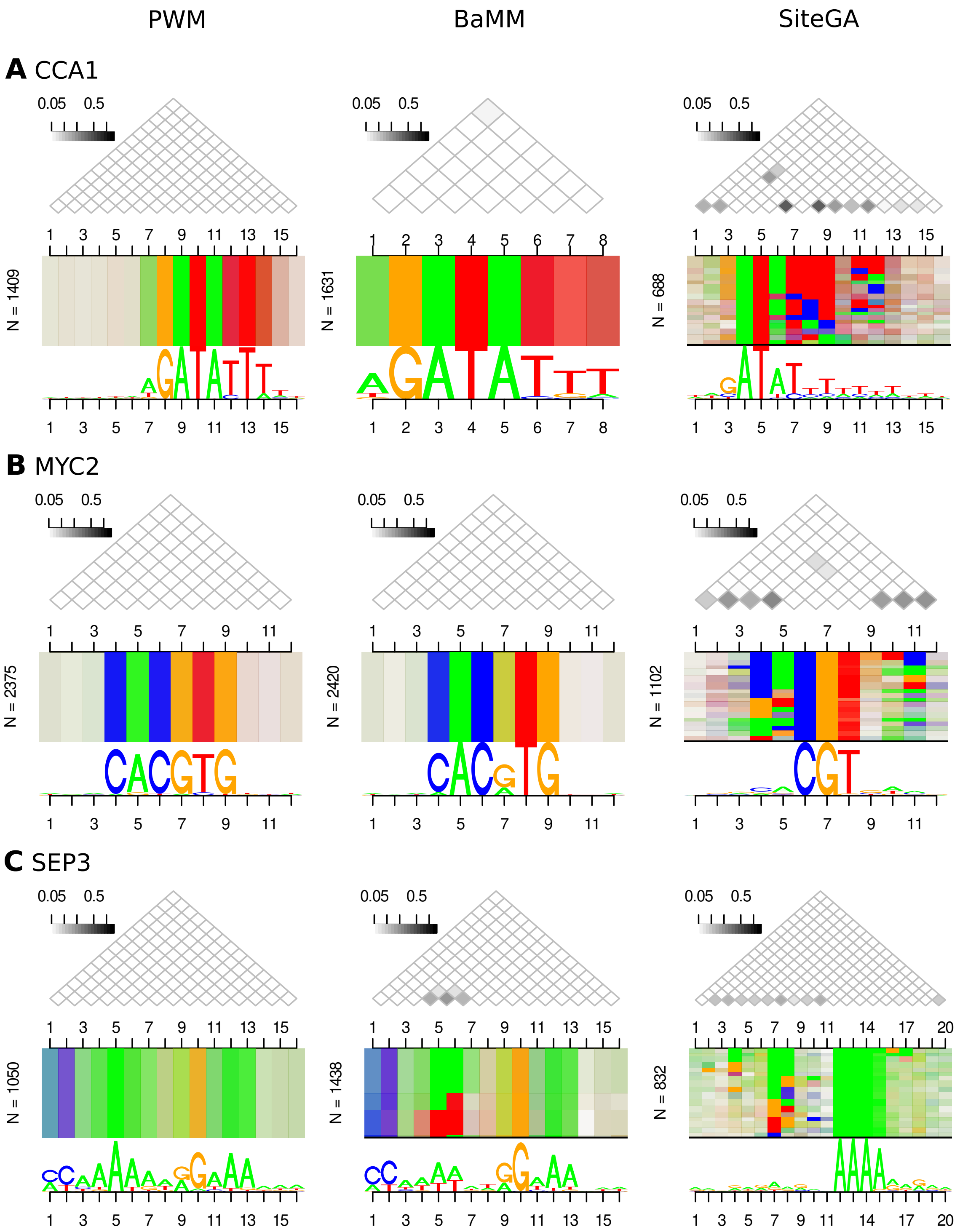

Figure S4. Traditional and alternative sequence logos representing the alignments of predicted BSs for datasets of CCA1 (**A**), MYC2 (**B**), and SEP3 (**C**) TFs. Three columns show PWM, BaMM, and SiteGA recognition models. In each of the 3x3 cells the traditional sequence logo is located under the alternative sequence logo (DepLogo, Grau et al., 2019). Above each alternative logo, the triangle matrix shows the mutual information as a measure of position interdependency. The dependencies are visualized as the horizontal boxes showing pairs of interacting nucleotides; from the left side of each logo the total number of BSs (N) is designated. For each model the peaks were sorted in the descending order of the recognition score. The alignments of predicted BSs were used to compute the logos.

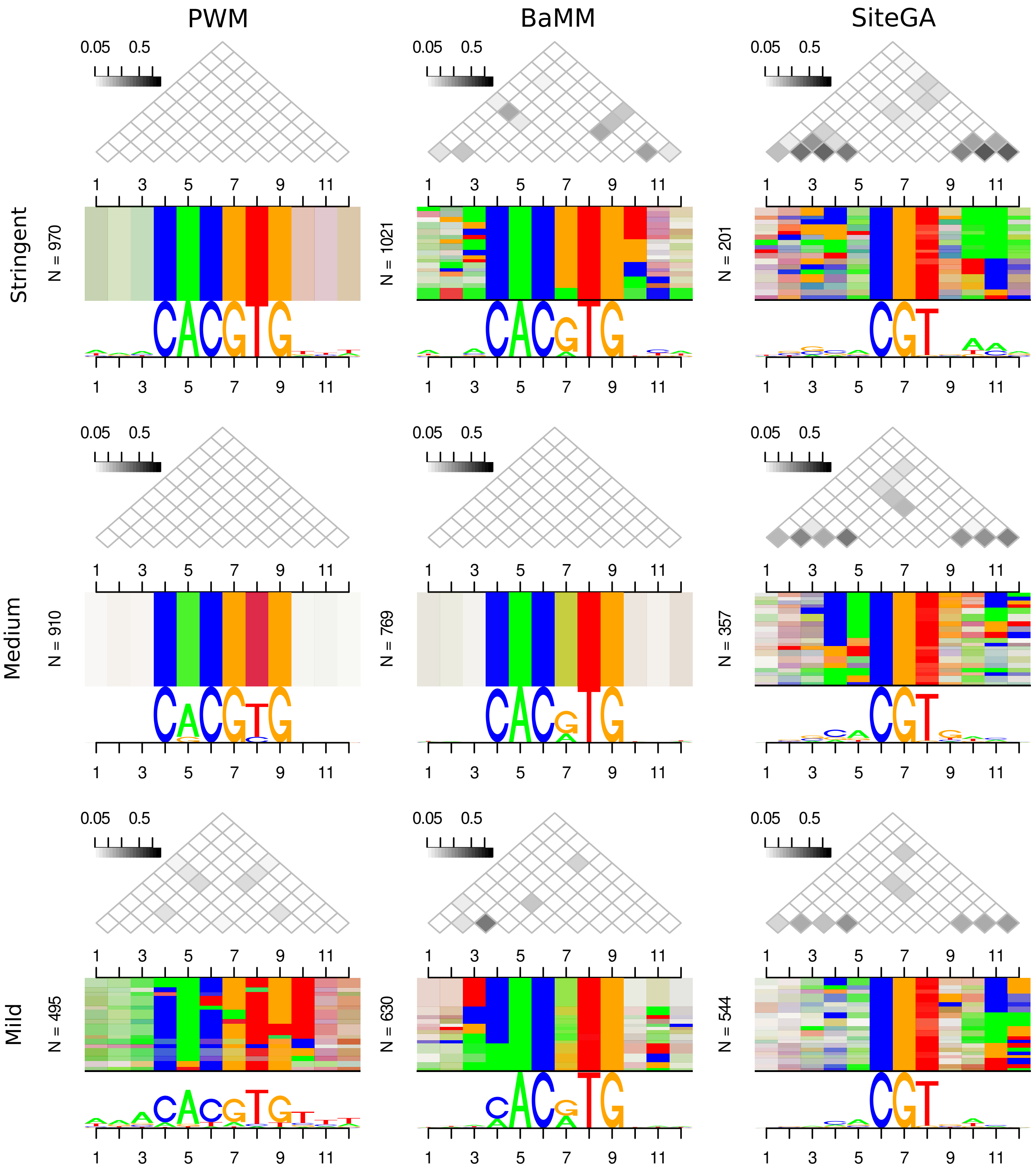

Figure S5. Traditional and alternative sequence logos representing the alignments of sites predicted by PWM, BaMM, and SiteGA recognition models for MYC2 TF. Three columns show three models. Three rows show the growth of the recognition score: the bottom, middle and top rows depict the mild, medium, and stringent ranges of recognition scores (2.5E-4 < ERR ⩽ 5E-4, 1E-4 < ERR ⩽ 2.5E-4, and ERR ⩽ 1E-4, respectively, see Materials and Methods). In each of the 3x3 cells the traditional sequence logo is located under the alternative sequence logo (DepLogo, Grau et al., 2019). Above each alternative logo, the triangle matrix shows the mutual information as a measure of the position interdependency. The dependencies are visualized as the horizontal boxes showing pairs of interacting nucleotides; from the left side of each logo the total number of BSs (N) is designated.

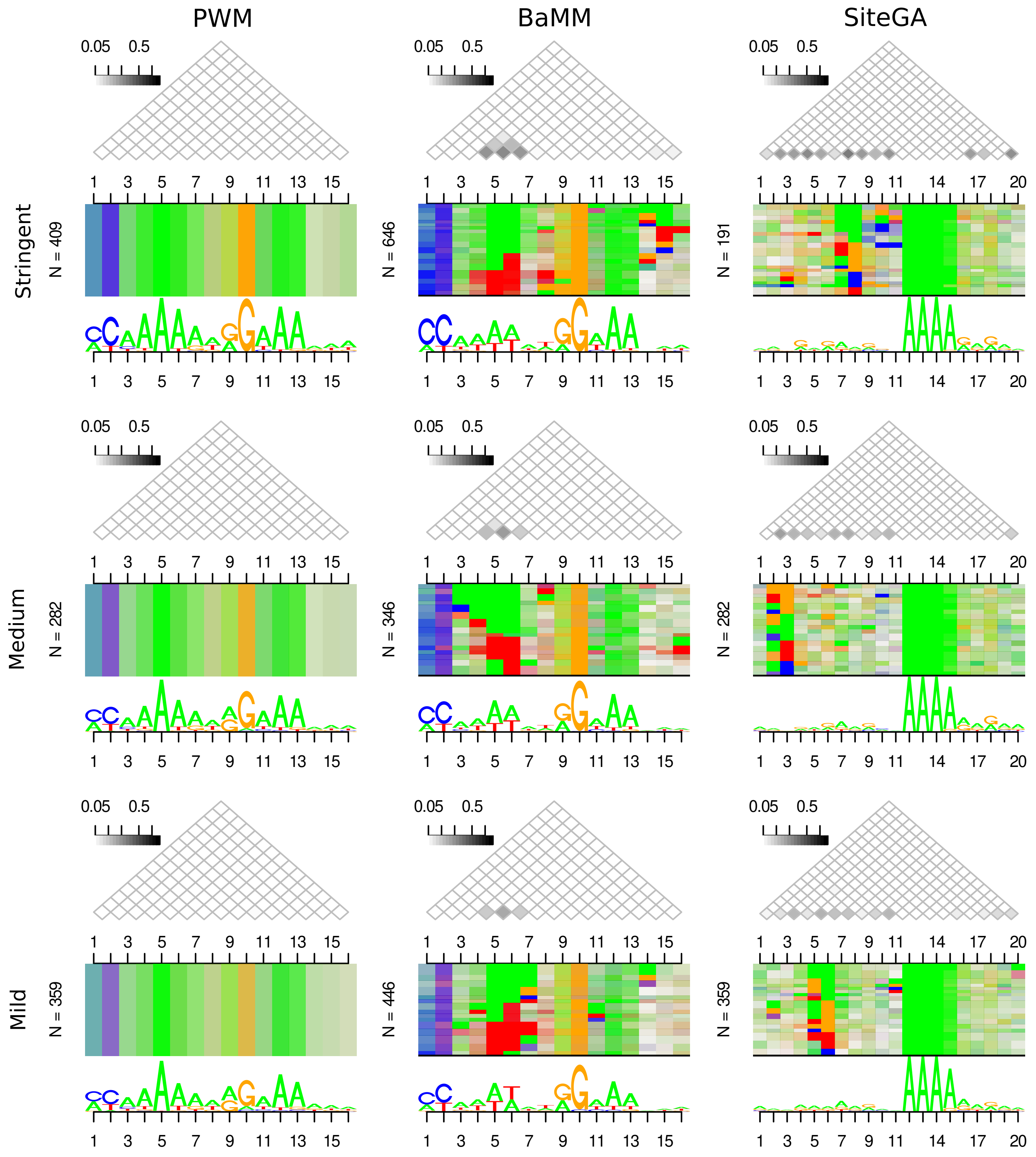

Figure S6. Traditional and alternative sequence logos representing the alignments of sites predicted by PWM, BaMM, and SiteGA recognition models for SEP3 TF. Three rows show the growth of the recognition score: the bottom, middle and top rows depict the mild, medium, and stringent ranges of recognition scores (2.5E-4 < ERR ⩽ 5E-4, 1E-4 < ERR ⩽ 2.5E-4, and ERR ⩽ 1E-4, respectively, see Materials and Methods). In each of the 3x3 cells the traditional sequence logo is located under the alternative sequence logo (DepLogo, Grau et al., 2019). Above each alternative logo, the triangle matrix shows the mutual information as a measure of the position interdependency. The dependencies are visualized as the horizontal boxes showing pairs of interacting nucleotides; from the left side of each logo the total number of BSs (N) is designated.

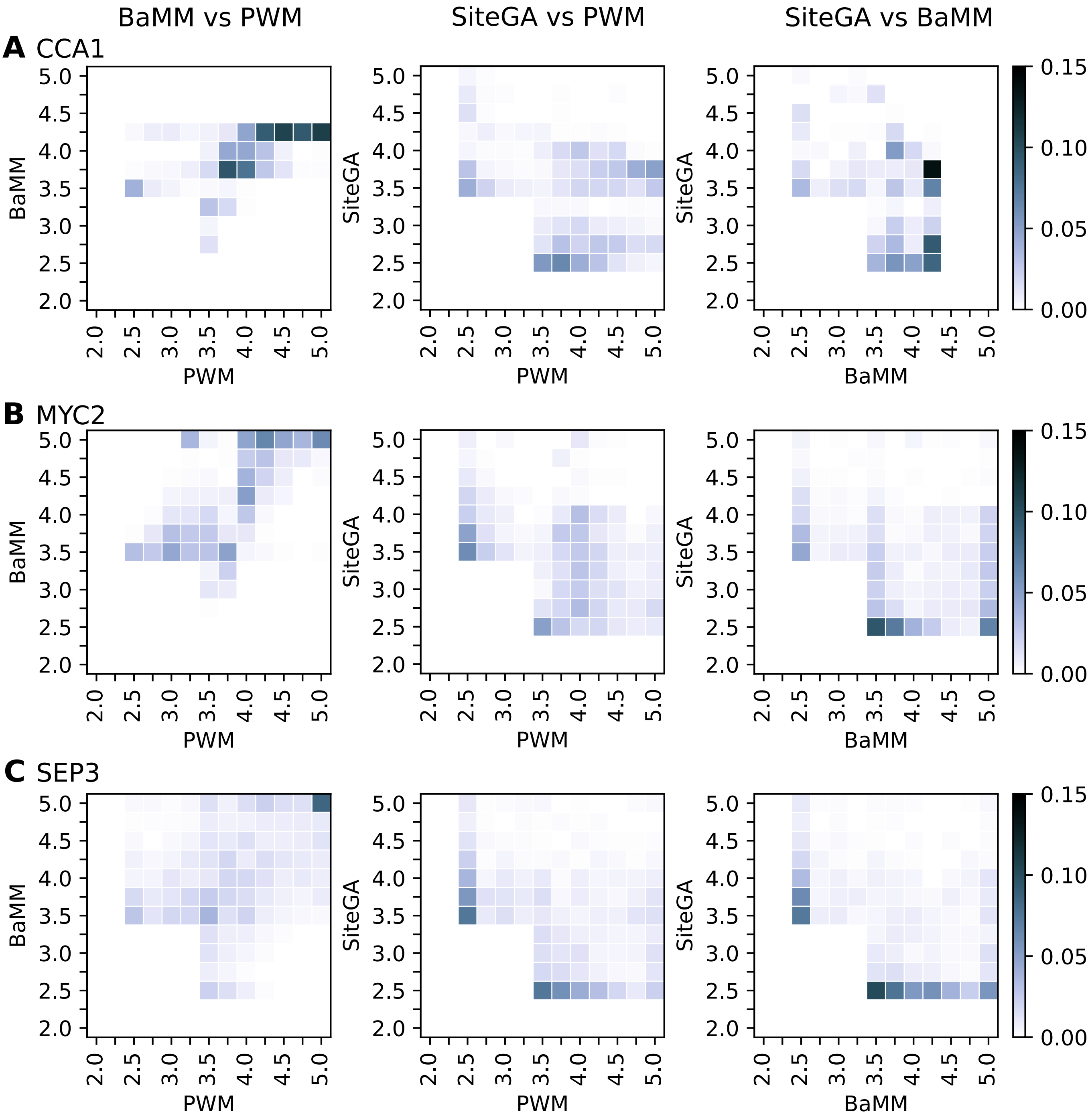

Figure S7. Abundance of the BSs possessing various recognition scores in distinct pairwise combinations of the models. Panels **(A)**, **(B)** and **(C)** represent the heatmap computed for the scores of BSs from the datasets for CCA1, MYC2, and SEP3 TFs, respectively. Rows and columns in each heatmap show the recognition score of the models as the logarithmic ERR, $-\mathrm{Log}_{10}(\mathrm{ERR})$. Colors denote the abundance of the BSs recognized by the models with the specific ERRs of two models. The maximal abundance 1 respects recognition of all BSs. Left, central, and right parts show combinations of models BaMM/PWM, SiteGA/PWM, and SiteGA/BaMM. For each pair of the motif models, we required that for at least one model a recognition score respected ERR $\leqslant$ 5E-4.

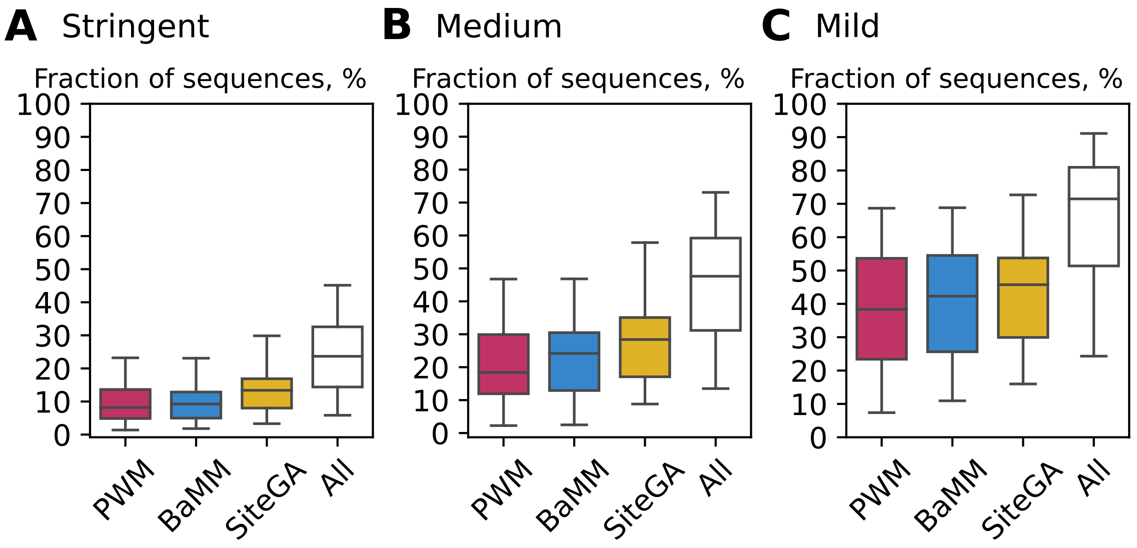

Figure S8. Comparison of application of PWM, BaMM, and SiteGA motif models and their combination for the joined background data respecting the benchmark collection of ChIP-seq datasets. Panels **(A)**, **(B)** and **(C)** show boxplots computed with the stringent, medium, and mild thresholds (recognition scores respecting ERR $\leqslant$ 1E-4, ERR $\leqslant$ 2.5E-4, and ERR $\leqslant$ 5E-4). Each boxplot shows the distribution of the fractions of sequences containing the motifs predicted by sole models, and the fraction of sequences containing the motifs predicted by at least one model out of three (white boxes 'All'). Red, blue, and yellow columns mark PWM, BaMM, and SiteGA models. The boxplots present distributions of the Q_1, Q_2 and Q_3 quartiles of the fractions of sequences. Whiskers below/above the Q_1/Q_3 respect minimum/maximum values if they were located within 1.5 interquartile range $(IQR = Q_3–Q_1)$ from Q_1/Q_3, otherwise they are equal to $\{Q_1 - 1.5 * IQR\}/\{Q_3 + 1.5 * IQR\}$, respectively. In the latter case, we marked all other points as outliers.

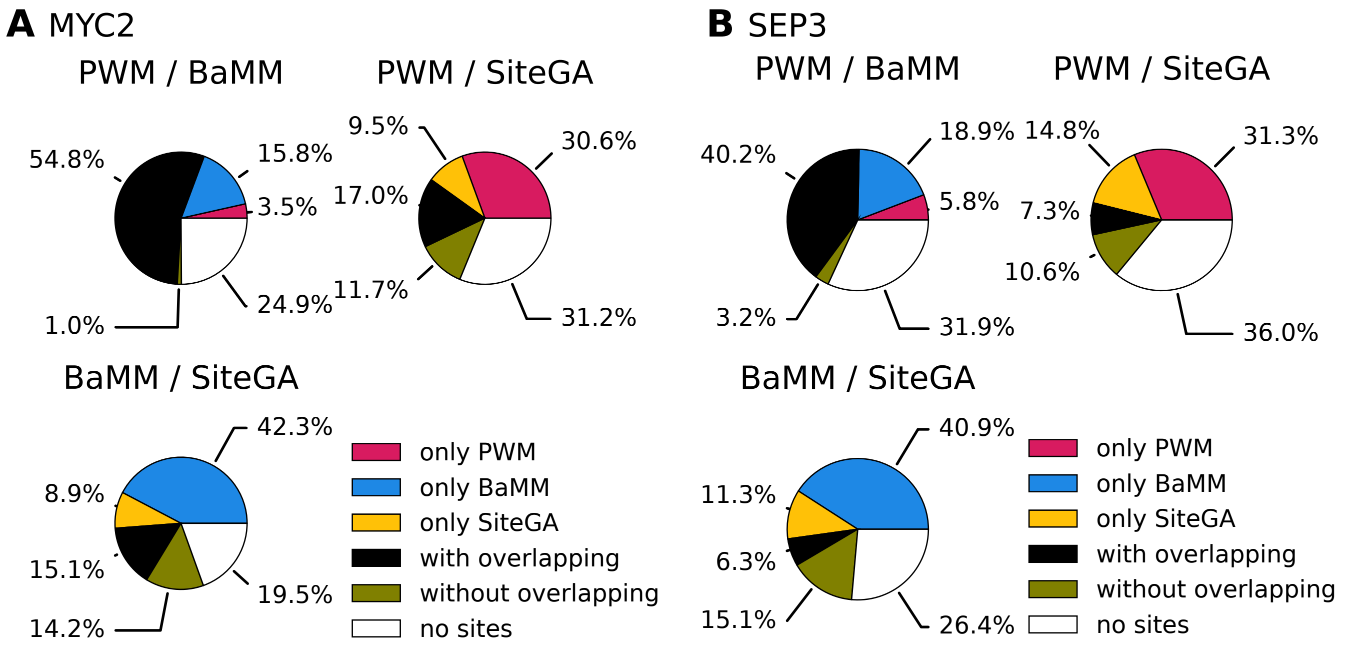

Figure S9. Classification of peaks from **(A)** MYC2 and **(B)** SEP3 datasets taking into account the presence of the motifs and the overlaps of their positions. In each panel three pie charts show pairwise combinations of PWM/BaMM, PWM/SiteGA, and BaMM/SiteGA models. Red, blue and yellow sectors mark the fractions of peaks recognized by only one model in pairs (PWM, BaMM, and SiteGA, respectively). Black/olive sectors denote the fractions of peaks recognized by two models with/without overlapping motifs. The white color means that the motifs of both models are absent. The analysis was performed with the medium threshold (recognition scores respecting ERR $\leqslant$ 2.5E-4).

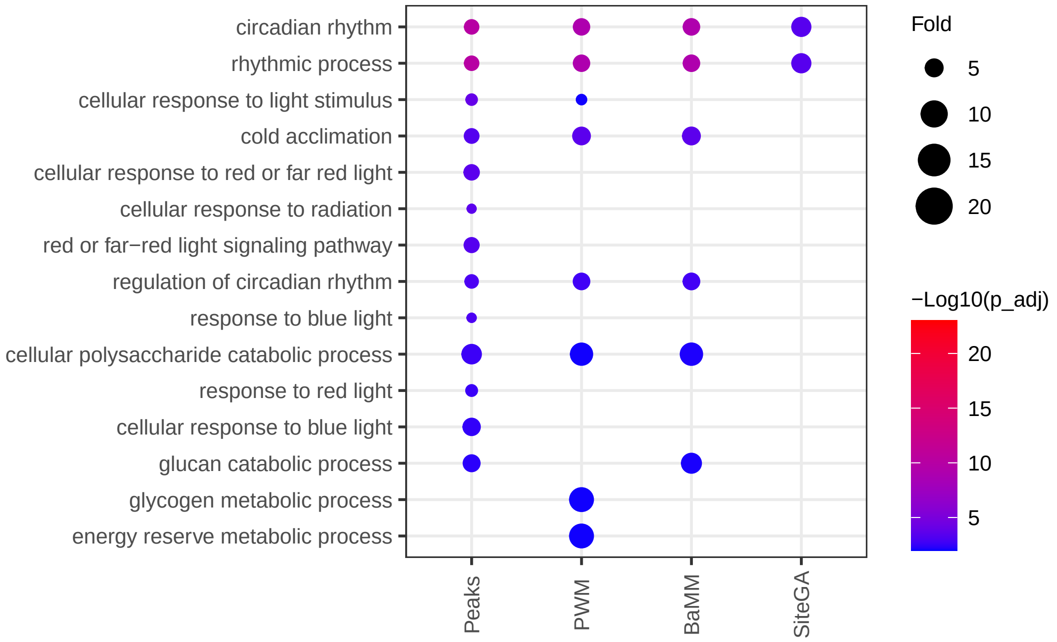

Figure S10. GO terms significantly enriched in the genes harboring the ChIP-Seq peaks from the CCA1 dataset with the BSs predicted by various recognition models. 2500 bp long upstream/downstream regions of the genes or entire genes should overlap a peak or its site Motif prediction was done with PWM, BaMM, and SiteGA models. Axis X denotes the motif models. Axis Y lists the enriched GO terms. The size of a circle implies the fold ratio, i.e. the ratio between the fractions of the genes possessing a GO term for a test list to that for the whole genome. The color represents the significance of a GO term enrichment (adjusted p-value, p_adj). Thresholds Fold $\geqslant 3$ and p_adj < 0.01 were applied to draw the heatmap.

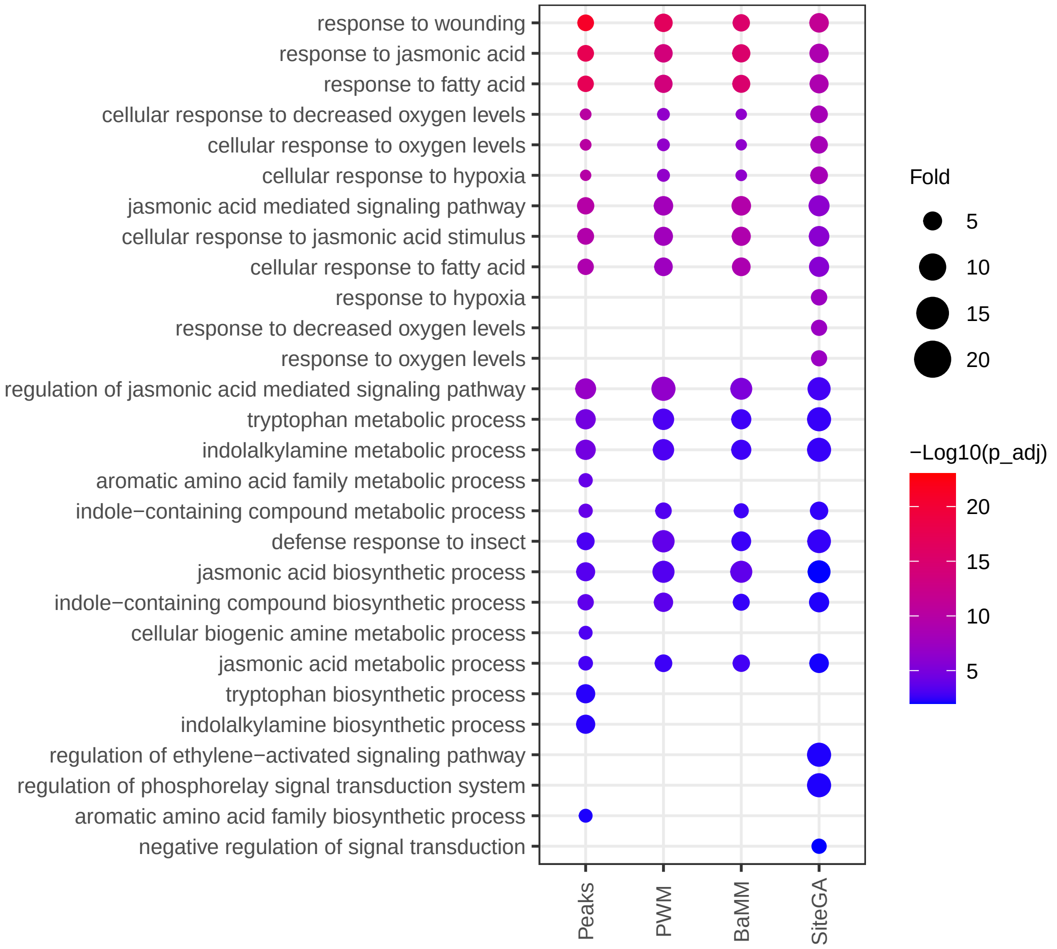

Figure S11. GO terms significantly enriched in the genes harboring ChIP-Seq peaks from the MYC2 dataset with motifs of various recognition models. 2500 bp long upstream/downstream regions of the genes or entire genes should overlap a peak or its site. Motif prediction was done with PWM, BaMM, and SiteGA models. Axis X denotes the motif models. Axis Y lists the enriched GO terms. The size of a circle implies the fold ratio, i.e. the ratio between the fractions of the genes possessing a GO term for a test list to that for the whole genome. The color represents the significance of a GO term enrichment (adjusted p-value, p_adj). Thresholds fold $\geqslant 3$ and p_adj < 0.01 were applied to draw the heatmap.

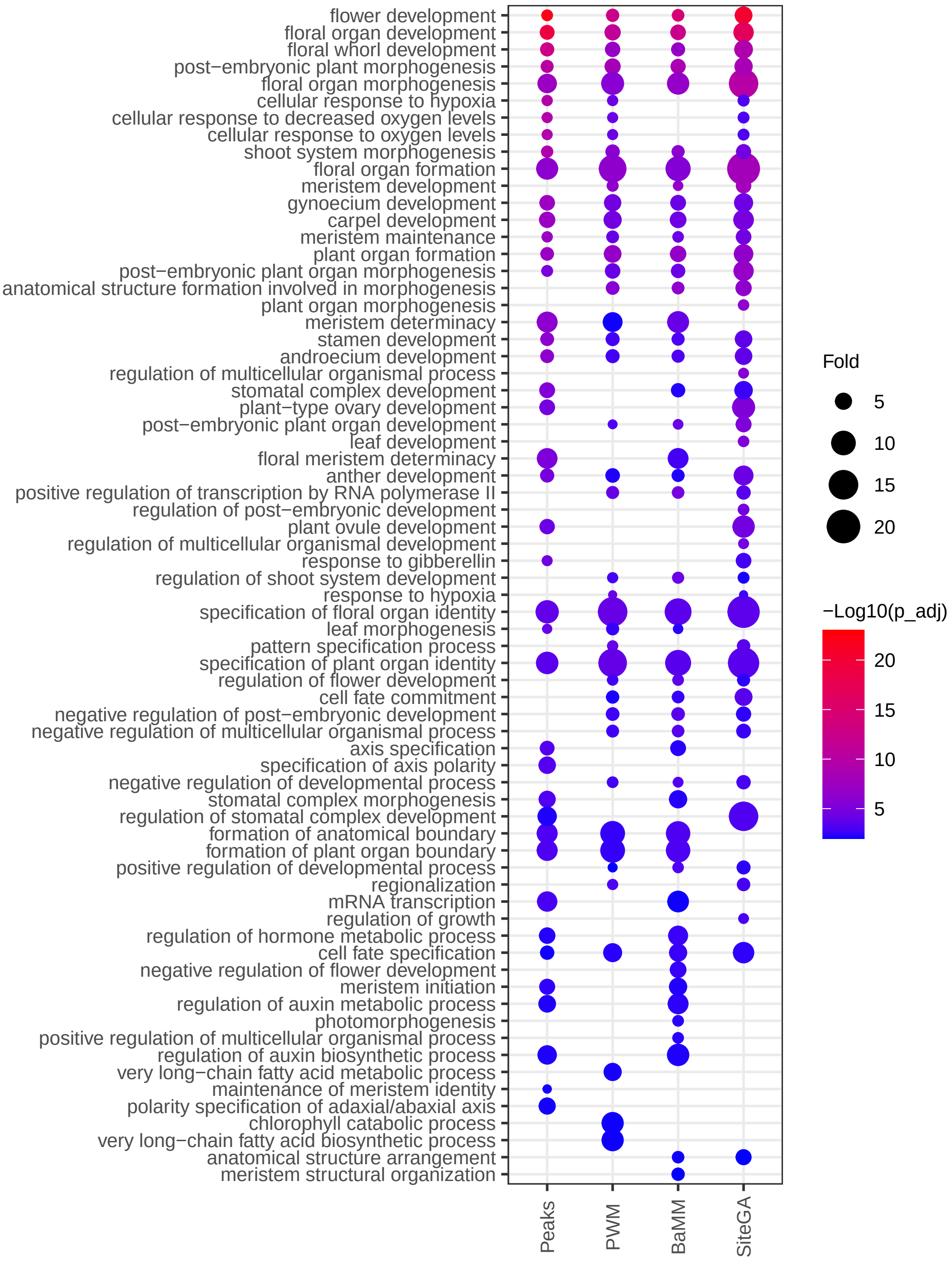

Figure S12. GO terms significantly enriched in the genes harboring ChIP-Seq peaks from the SEP3 dataset with motifs of various recognition models. 2500 bp long upstream/downstream regions of the genes or entire genes should overlap a peak or its site. Motif prediction was done with PWM, BaMM, and SiteGA models. Axis X denotes the motif models. Axis Y lists the enriched GO terms. The size of a circle implies the fold ratio, i.e. the ratio between the fractions of the genes possessing a GO term for a test list to that for the whole genome. The color represents the significance of a GO term enrichment (adjusted p-value, p_adj). Thresholds fold $\geqslant 3$ and p_adj < 0.01 were applied to draw the heatmap.
