# Supplementary material for: Motif models proposing independent and interdependent impacts of nucleotides are related to high and low affinity transcription factor binding sites in Arabidopsis
Source: Front Plant Sci. 2022 Jul 28;13:938545. doi: 10.3389/fpls.2022.938545 (PMC9373801; doi:10.3389/fpls.2022.938545)
Supplement: Supplementary file 2 [file Data_Sheet_2.PDF]

Supplementary Material

1 SUPPLEMENTARY METHODS

1.1 SiteGA algorithm

Input data of SiteGA de novo motif search comprise the foreground dataset consisting of N sequences of peaks and the background datasets of sequences with the size at least several times larger than that of the foreground dataset. The SiteGA model describes the motifs with the set of M locally positioned dinucleotides (LPDs). The model deduces mutual dependencies of frequencies of these LPDs to build the recognition function with the linear discriminant analysis approach. The SiteGA model uses the genetic algorithm (GA) to optimize the population of LPDs or 'individuals'. GA ranked the individuals in the population in the descending order of the fitness function. Evolution of population implies multiple consecutive iterations. An iteration includes (a) multiple mutation attempts for each individual, and (b) respective number of recombination attempts for various pairs of individuals. A mutation changes one or several characteristics within an individual, while a recombination exchanges them between two distinct individuals. The success of a mutation or recombination implies the growth of the fitness function. We stop GA, if for one iteration the first-ranked individual of a population shows too small increment of the fitness function, or if the numbers of mutations and recombinations become too small. The first ranked individual represents the final SiteGA model. The SiteGA recognition function we defined as described earlier (26).

Each individual comprises two sets of characteristics: (a) the set of LPDs $\{s_m, e_m, d_m\}(1 \leqslant m \leqslant M)$ located within potential sites of the model's length W bp, (b) positions pn and orientations on of sole sites in all peaks $(1 \leqslant n \leqslant N)$. Here $[s_m, e_m, d_m]$ mean a location and dinucleotide type of LPD_m $(1 \leqslant s_m, e_m < W,\ 0 \leqslant s_m - e_m \leqslant L_{max}{-}1,\ 0 \leqslant d_m \leqslant 15)$, the constants L_max and 15 refer to the default maximal length of LPD and the number of all dinucleotides 16. We initiate a population of individuals with randomly chosen LPDs and motifs in peaks. Positions $\{p_n\}$ and orientations $\{o_n\}$ define an alignment of motifs in peaks, $X = \{x[1], x[2], \ldots, x[N]\}$. The fitness function $F(X)$ for this alignment implies its quality in GA as follows:

$$F(X) = D(X) * E(X) \tag{S1}$$

Here the first factor $D(X)$ reflects the dependencies between positions within an alignment, while the second one $E(X)$ implies its enrichment. The former is the Mahalanobis distance between average LPD frequencies for foreground and background datasets ($f_i^{(1)}$ and $f_i^{(2)}$),

$$D(X) = \sum_{i=1}^{M}\sum_{j=1}^{M}[\{f_i^{(1)}(X) - f_i^{(2)}\} * S_{i,j}^{-1} * \{f_j^{(1)}(X) - f_j^{(2)}\}] \tag{S2}$$

Here $S_{i,j}^{-1}$ denotes the inverse matrix for the sum of covariation matrices respecting $f_i^{(1)}$ and $f_i^{(2)}$ vectors. We estimate the covariance matrix for the vector $\{f_i^{(2)}\}$ as diagonal and compute it through dispersions of LPD frequencies for the background dataset. The factor $E(X)$ means the average fold enrichment of the k-mers of specific length Z within an alignment. This fold reflects the difference between foreground

and background datasets. As preliminary steps to GA, (a) we count enrichment log-ratios of all 4^Z k-mers, $R_1(t) = Log_{10}[\frac{p_t^{(1)}}{p_t^{(2)}}]$, here the index t implies the type of k-mer $(1 \leqslant t \leqslant 4^Z)$, $p_t^{(1)}$ and $p_t^{(2)}$ denote frequencies of t-th k-mer for foreground and background datasets; (b) we mark all positions of motifs in peaks with respective enrichment log-ratios, $R_W(x[n]) = \frac{1}{W-Z+1}\sum_{t=x[n]}^{W-Z+x[n]} R_1(t)$. Hence, the factor $H(X)$ means the enrichment of k-mers within an alignment,

$$Log_{10}\{E(X)\} = \frac{1}{N}\left\{\sum_{n=1}^{N} R_W(x[n])\right\} = \frac{1}{N(W-Z+1)}\sum_{n=1}^{N}\left\{\sum_{t=x[n]}^{W-Z+x[n]} Log_{10}\left[\frac{p_t^{(1)}}{p_t^{(2)}}\right]\right\} \quad \text{(S3)}$$

The default maximal LPD length L_{max} is 6, and the length Z of k-mers for enrichment estimation is 6. The parameters ‘Number of LPDs’ M and ‘Motif length’ W we adopted by the bootstrap cross-validation procedure, the best ones respect to the maximal pAUC measure.
